# Rapid‐Onset Therapeutic Effects of Delta Opioid Receptor Agonists on Depression‐Like Behaviors Induced by Chronic Social Defeat Stress

**DOI:** 10.1002/npr2.70059

**Published:** 2025-09-29

**Authors:** Akihisa Tokuda, Yasuyuki Nagumo, Keita Kajino, Keita Iio, Katsuyasu Sakurai, Seiya Mizuno, Akiyoshi Saitoh, Tsuyoshi Saitoh, Hiroshi Nagase

**Affiliations:** ^1^ International Institute for Integrative Sleep Medicine (IIIS) University of Tsukuba Tsukuba Ibaraki Japan; ^2^ Doctoral Program in Medical Sciences, Graduate School of Comprehensive Human Sciences University of Tsukuba Tsukuba Japan; ^3^ Degree Programs in Pure and Applied Sciences, Graduate School of Science and Technology University of Tsukuba Tsukuba Japan; ^4^ Laboratory Animal Resource Center and Trans‐Border Medical Research Center University of Tsukuba Tsukuba Japan; ^5^ Laboratory of Pharmacology, Faculty of Pharmaceutical Sciences Tokyo University of Science Noda Japan; ^6^ Institute of Medicine University of Tsukuba Tsukuba Japan

**Keywords:** anxiety, chronic social defeat stress, depression, opioid delta receptor agonist, social behavior

## Abstract

Depression is prevalent, yet conventional antidepressants often require time to produce therapeutic effects. Therefore, novel antidepressants with a more rapid onset of action are urgently needed. This study investigated the potential of δ opioid receptor (DOP) agonists as fast‐acting antidepressants, comparing their efficacy with that of existing treatments. We utilized the chronic social defeat stress (CSDS) model, which closely mimics clinical depressive symptoms, to evaluate the therapeutic effects of DOP agonists (SNC80, KNT‐127) and paroxetine, a selective serotonin reuptake inhibitor. Mice exposed to CSDS for 10 days showed social avoidance toward aggressor mice along with generalized depression and anxiety‐like behaviors. Both DOP agonists demonstrated dose‐dependent therapeutic effects at 1–10 mg/kg and showed improvements within 10–14 days of chronic administration. In contrast, paroxetine alleviated these symptoms after 28 days of treatment. These findings suggest that DOP agonists may offer a faster‐acting alternative to current antidepressant therapies.

## Abstract 2

Delta opioid receptor agonists improved social avoidance, depression, and anxiety‐like symptoms induced by chronic social defeat stress within 14 days of chronic administration. Furthermore, the therapeutic effects were observed earlier than with the existing antidepressant paroxetine (28 days).

## Graphical abstract

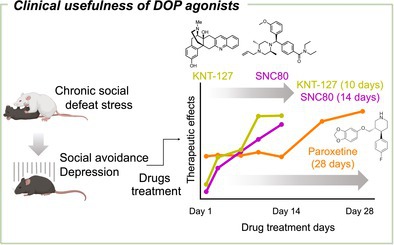

## 1 Introduction

Depression is frequently triggered by the chronic buildup of daily life stressors [1, 2]. Its lifetime prevalence is 15%–18%, affecting nearly one in five people [2]. Beyond its impact on mental health, depression disrupts physical health, work productivity, and social relationships, underscoring its significant societal impact. A major concern with depression is its tendency to become chronic, with an approximately 80% recurrence rate [2]. Furthermore, chronic depression significantly heightens suicide risk, contributing to 534.3 deaths per 100 000 individuals, making it one of the leading causes of suicide globally [3]. Given this context, achieving rapid and effective treatment of depression remains a critical objective in psychiatry [4].

Commonly prescribed antidepressants include tricyclic antidepressants (TCAs), selective serotonin reuptake inhibitors (SSRIs), serotonin–noradrenaline reuptake inhibitors (SNRIs), and noradrenergic and specific serotonergic antidepressants (NaSSAs) [5]. However, these drugs often cause serious side effects, such as sexual dysfunction, weight gain, nausea, diarrhea, and headaches [6]. Additionally, they typically require a treatment duration of at least 3–4 weeks for therapeutic benefits, during which the risk of suicide may increase due to the delayed onset of action. This delay underscores an urgent need for new antidepressants with fewer adverse effects and faster onset to enhance patient safety and treatment efficacy [4].

The δ opioid receptor (DOP) has recently emerged as a promising alternative target for antidepressant development [7]. DOP, a Class A G‐protein‐coupled receptor (GPCR), is highly expressed in brain regions associated with emotional regulation, including the insula cortex, prefrontal cortex, cingulate cortex, amygdala, striatum, hippocampus, and nucleus accumbens [8, 9]. DOP agonists have shown antidepressant effects even after a single administration in rodent studies [10, 11], suggesting they may work through a different mechanism than conventional medications, potentially offering quicker therapeutic effects and fewer side effects. However, research on the therapeutic potential of DOP agonists for clinically relevant symptoms of depression is scarce.

In this study, we investigated the antidepressant efficacy of DOP agonists using the chronic social defeat stress (CSDS) model [12], a well‐established model that replicates key aspects of depression's etiology and pathophysiology [13, 14]. The CSDS model is widely valued for its alignment with core animal model criteria (face validity, construct validity, and predictive validity) in psychiatric research, making it a powerful tool for assessing therapeutic interventions closely resembling human conditions. Our experiments evaluated two representative DOP agonists: SNC80, a commonly studied compound in DOP pharmacology, and KNT‐127, a potent agonist known for its high selectivity and receptor affinity [15]. The goal was to determine whether chronic administration of these DOP agonists could alleviate depression‐like behaviors induced by chronic stress, thereby highlighting their potential as rapid‐acting antidepressants with fewer side effects compared to conventional treatments.

## 2 Methods

### 2.1 Animals

All animal experiments were approved by the Institutional Animal Care and Use Committee of the University of Tsukuba (approval no. 18‐178, 19‐309, 20‐287, 20‐289, 21‐313, 21‐321, 22‐355, 23‐282, 24‐061), and all procedures were conducted following the University's Guidelines for Animal Experiments. Male C57BL/6J mice (5 weeks old) and retired breeder male ICR mice were obtained from Japan SLC (Tokyo, Japan). Mice were maintained under specific pathogen‐free conditions with a 12‐h light/dark cycle (lights on from 09:00 to 21:00) at an ambient temperature of 23°C ± 1°C.

### 2.2 Generation of *Oprd1*‐Cre Mice

CRISPR‐Cas9 technology was used to generate *DOP‐Cre* knock‐in mice. The CRISPR target sequence containing the PAM sequence (5′‐AGA CGG ACA CGG CGG CGC CAT GG‐3′) was selected to integrate the nuclear translocation signal (NLS)‐cre sequence within exon 1 of the *Oprd1* gene as the sgRNA target. We purchased synthetic cDNA containing this target in sequence from Integrated DNA Technologies. A chimeric intron, NLS‐cre, and rabbit globin polyadenylation sequences were inserted between the 5′ and 3′ homology arms of the donor DNA, resulting in the generation of *pOprd1‐CI‐Cre* donor plasmid. The 5′ and 3′ homology arms in the donor DNA were designed based on the genomic regions chr4:132145867–132144386 and chr4:132144382–132143000 (GRCm38/mm10), respectively. Pregnant mare serum gonadotropin (5 units) and human chorionic gonadotropin (5 units) were intraperitoneally administered to female C57BL/6J mice in a 48‐h interval, followed by mating with male C57BL/6J mice. Zygotes were retrieved from the oviducts of mated female mice, and the CRISPR‐Cas9 ribonucleoprotein complex and the donor plasmid DNA were microinjected into the zygotes following a previously reported protocol [16]. The microinjected zygotes were transplanted into the oviducts of pseudo‐pregnant ICR females, resulting in the birth of offspring. The homozygous *Oprd1*‐Cre mice, in which Cre was inserted into exon 1 of the *Oprd1* gene, functioned as the *Oprd1* knockout mice (Figure S1, hereafter referred to as DOP KO mice).

### 2.3 In Situ Hybridization

Mice were transcardially perfused, first with 10% sucrose in 0.1 M phosphate‐buffered saline (PBS, pH 7.4) to flush out the blood, followed by 4% paraformaldehyde (PFA). Brains were collected and postfixed overnight in 4% PFA at 4°C. After fixation, brains were cryoprotected in 30% sucrose until they sank, embedded in OCT compound (Sakura Finetek), and coronally sectioned at 40 μm thickness using a cryostat (Leica Biosystems, Nussloch, Germany).

Hybridization Chain Reaction (HCR) RNA‐FISH was performed to detect DOP transcripts using reagents from Molecular Instruments. In brief, sections were washed in DEPC‐treated PBS and treated with proteinase K (Roche Applied Science, Germany) at 37°C for 30 min. After preincubation in probe hybridization buffer at 37°C for 30 min, sections were hybridized overnight at 37°C with DOP‐specific probes (Molecular Instruments). Following washes with HCR probe wash buffer and 5× SSCT, sections were incubated overnight at 25°C with hairpins conjugated to Alexa Fluor 546 to amplify and visualize the hybridization signals. After washing with 2× SSC, sections were counterstained with 4′,6‐diamidino‐2‐phenylindole (DAPI) (Dojindo, D523), washed again, mounted, and coverslipped. Imaging was performed using an LSM800 confocal microscope (Carl Zeiss AG, Oberkochen, Germany).

### 2.4 Drugs

(6*R*,6a*S*,14a*R*)‐17‐methyl‐5,6,7,14‐tetrahydro‐6aH‐6,14a‐(epiminoethano)‐naphtho[2,1‐b]acridine‐2,6a‐diol (KNT‐127·2 HCl in house [15]), (+)‐4‐[(α*R*)‐α‐((2*S*,5*R*)‐4‐Allyl‐2,5‐dimethyl‐1‐piperazinyl)‐3‐methoxybenzyl]‐*N*,*N*‐diethylbenzamide (SNC80; Cayman, MI, USA), and (−)‐(3*S*,4*R*)‐4‐(4‐fluorophenyl)‐3‐[(3,4‐methylenedioxy)phenoxymethyl]piperidine (paroxetine·HCl; Tokyo Chemical Industry Co. Ltd., Tokyo, Japan) were studied. SNC80 was dissolved in 1 M HCl (Kanto Chemical Co., Tokyo, Japan) and diluted with saline (Otsuka Pharmaceutical Factory, Tokushima, Japan) to a final concentration of 0.3% HCl. KNT‐127 and paroxetine were dissolved in saline.

### 2.5 Social Defeat Stress

Social defeat stress (SDS) was conducted for 10 days as previously described [12, 17], with minor modifications. Before CSDS, male C57BL/6J mice were isolated for 1 week with ad libitum access to food and water. Retired male ICR mice (aggressor mice) were prescreened for aggressiveness toward C57BL/6J mice, and those showing high aggressiveness were used for CSDS. During CSDS, a C57BL/6J mouse was introduced to the compartment housing an aggressor for 10 min. Following this exposure, the defeated C57BL/6J mouse was placed in the adjacent compartment, separated by a metal mesh partition, where it remained overnight. Each C57BL/6J mouse faced a different aggressor each day. To prevent excessive physical harm, stress exposure was stopped if the aggressor continued biting the C57BL/6J mouse's back for more than approximately 15 s.

### 2.6 Social Interaction Test

**FIGURE 1:**
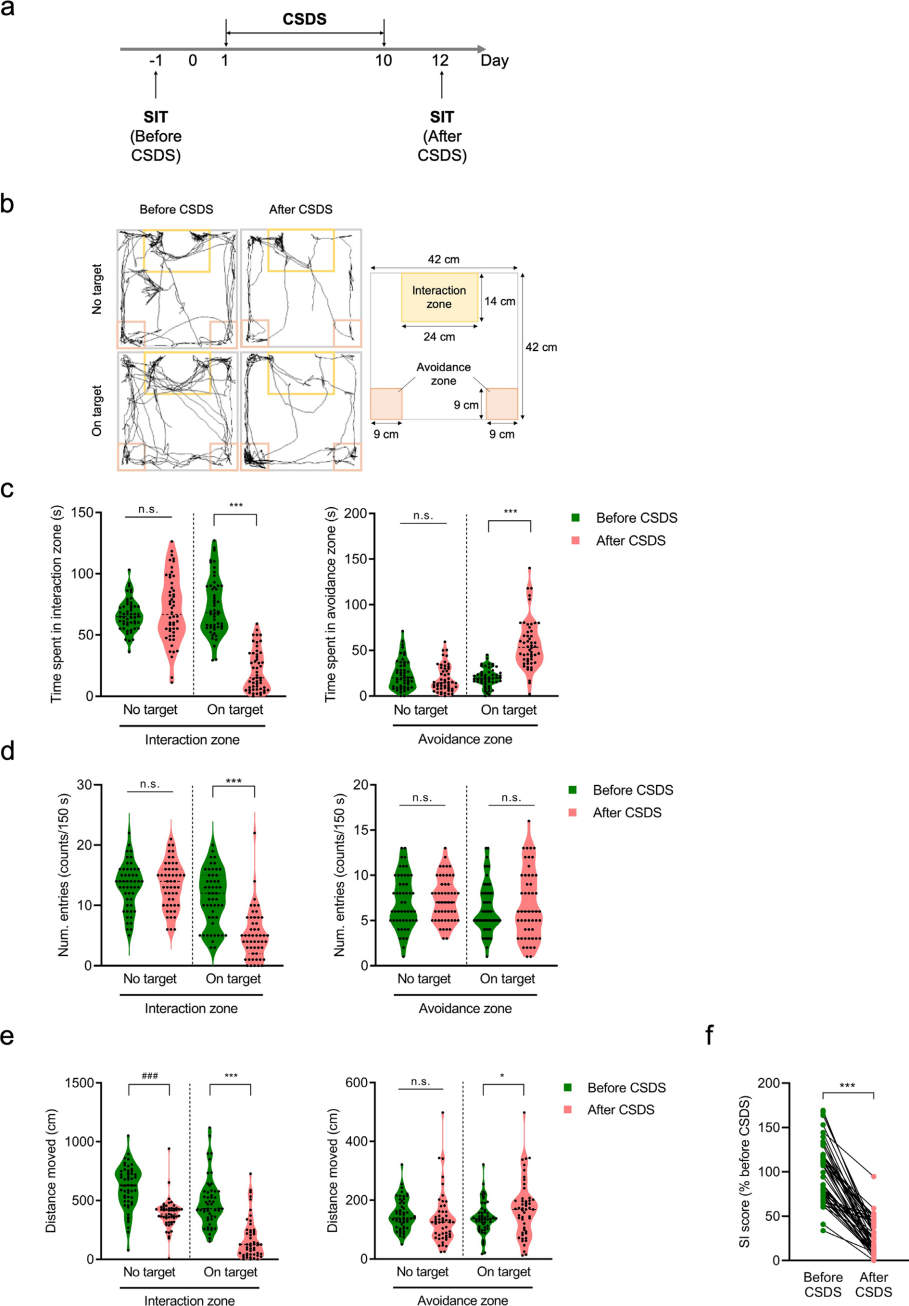
Social avoidant behaviors induced by CSDS. (a) Experimental timeline of the chronic social defeat stress (CSDS) procedure and the social interaction test (SIT). CSDS was administered for 10 consecutive days, followed by SIT to assess social avoidance behaviors in stressed mice (*n* = 52). (b) (Right) Representative tracking images from SIT sessions before and after CSDS in stressed mice. (Left) Diagram of the recording chamber showing the social interaction and avoidance zones. (c) Violin plots depicting time spent by stressed mice in the interaction zone (left) or avoidance zone (right) during “no target” and “on target” SIT sessions. Data represent the mean. ***p < 0.001, paired Student's *t*‐test; n.s., not significant. (d) Violin plots of the mean number of entries into the interaction zone (left) and avoidance zone (right) during “no target” and “on target” SIT sessions. Data represent the mean for 52 mice. ***p < 0.001, paired Student's *t*‐test; n.s., not significant. (e) Violin plots illustrating the mean total distance traveled in the interaction zone (left) and avoidance zone (right) during “no target” and “on target” sessions. Data represent the mean for 52 mice. *p < 0.05 and ***p < 0.001 versus before CSDS in “on target” sessions; ^###^ *p* < 0.001 versus before CSDS in “no target” sessions; paired Student's *t*‐test; n.s., not significant. (f) Changes in social interaction (SI) score before and after CSDS. SI score was calculated by dividing the time spent in the interaction zone during “on target” sessions by that spent in “no target” sessions, normalized to the average pre‐CSDS score. ***p < 0.001, paired Student's *t*‐test.

The social interaction test (SIT) assessed social avoidance behaviors, following a modified protocol [12, 18]. A defined zone (24 cm × 14 cm) near a grid enclosure at one wall of the open‐field chamber (42 cm × 42 cm × 42 cm) was marked as the interaction zone, while two square zones (9 cm × 9 cm) at the opposite wall were designated as avoidance zones (Figure 1). After habituating to the test environment for at least 1 h, a stressed control mouse was placed at the center of the open‐field chamber, facing away from the interaction zone, and allowed to move freely for 150 s in the absence of an aggressor (“no target” session). Following a brief (approx. 1 min) return to the home cage, the mouse was reintroduced to the chamber, this time with an aggressor in the grid enclosure, and allowed to move freely for 150 s (“on target” session).

Behavior was video‐monitored, and movement was tracked using the SMART video tracking system (Harvard Apparatus, Holliston, MA, USA). Metrics included entries and time spent in each zone, along with the total distance traveled. The social interaction (SI) score was calculated as follows:

$$\mathrm{SI}\;\text{score}=\frac{{\text{time spent in interaction zone in}}^{“}\mathrm{on}\;{\text{target}}^{”}\;\text{session}\;\left(s\right)}{{\text{time spent in interaction zone in}}^{“}\mathrm{no}\;{\text{target}}^{”}\;\;\text{session}\;\left(s\right)}\times 100$$

In this study, we evaluated the effects of repeated DOP agonist administration on CSDS‐induced behavioral changes, characterized by social avoidance behaviors accompanied by depression‐like symptoms. To evaluate the therapeutic effects of DOP agonists and exclude the influence of acute effects of DOP agonists, SIT was performed at least 18 h after administration.

### 2.7 Tail Suspension Test

Depressive‐like behaviors were assessed using the tail suspension test (TST), based on previously described methods with minor adjustments [19]. The test apparatus consisted of white acrylic walls (12.5 × 15 × 50 cm) with one open wall to allow for video recording. Following exposure to the CSDS protocol, mice were acclimated for at least 1 h in a room with an illuminance of 20 lx prior to the TST. During the test, each mouse's tail was threaded through a 4‐cm acrylic pipe to prevent climbing. Vinyl tape (12 cm in length) was wrapped around the mouse's tail, 1 cm from the end, with the opposite end of the tape attached to the apparatus ceiling to suspend the mouse upside down, ensuring the head was 20 cm above the ground. Mouse behaviors were video‐monitored for 6 min, with immobility time during the final 5 min recorded as an indicator of depressive‐like behavior.

### 2.8 Open‐Field Test

Anxiety‐like behaviors were evaluated using the open‐field test (OFT), based on previously established methods with minor modifications [20]. Isolated C57BL/6J mice were acclimated to the testing room with an illuminance of 20 lx for at least 1 h. Mice were then placed in an acrylic open‐field chamber (42 × 42 × 42 cm) and allowed to explore freely for 10 min. A video camera positioned above the chamber recorded the mice's movements, which were analyzed post‐test using the SMARTV3.0 video tracking system (Panlab, S.L., Barcelona, Spain). Time spent in the center zone (22 × 22 cm) and total distance traveled within the chamber were measured.

### 2.9 Statistical Analysis

Data are presented as mean ± SEM. Comparisons between the two groups were analyzed using either paired or unpaired *t*‐tests. For comparisons involving more than two groups, one‐way ANOVA was performed, followed by Tukey's test when statistical significance was detected. Using Python for *k*‐means clustering, CSDS mice were classified based on the time spent in the avoidance zone and interaction zone during the SIT conducted before and after CSDS exposure; the optimal number of clusters tested ranged from one to two clusters.

## 3 Results

### 3.1 Social Avoidance Behaviors Induced by CSDS

At the start of this study, mice exposed to CSDS were assessed for changes in social avoidance behavior using a social interaction test (Figure 1a,b). During the no‐target session, where no aggressor mouse was present in the interaction zone, there were no significant differences in time spent or the number of entries in either the interaction or avoidance zones for mice before and after CSDS exposure (Figure 1c,d). However, during the on‐target session, where an aggressor mouse was present, the time spent and the number of entries in the interaction zone were significantly reduced in mice after CSDS compared to before exposure (*p* < 0.001, paired *t*‐test; Figure 1c,d). In contrast, time spent in the avoidance zone during the on‐target session significantly increased after CSDS, although the number of entries into the avoidance zone did not (time spent in the avoidance zone: *p* < 0.001, paired *t*‐test; number of entries: not significant; Figure 1c,d).

We next performed cluster analysis on time spent in avoidance or interaction zone from stressed mice, both before and after CSDS. The measured mice were classified as belonging to clusters with low scores in the avoidance zone and high scores in the interaction zone (cluster B), and clusters with high scores in the avoidance zone and low scores in the interaction zone (cluster A) (Figure S2). Most mice in the on‐target session before CSDS belonged to cluster B (90.4%, 47 of 52), while the majority of mice in the on‐target session after CSDS were classified in cluster A (98.1%, 51 of 52). With a classification accuracy rate exceeding 90%, these cluster distinctions suggest that a significant number of mice exhibited clear social avoidance behaviors following CSDS.

Furthermore, after CSDS exposure, the total distance traveled by mice in the interaction zone significantly decreased, while the distance traveled in the avoidance zone significantly increased compared to before CSDS (interaction zone: *p* < 0.001; avoidance zone: *p* < 0.05, paired *t*‐test; Figure 1e). To further quantify social interaction levels with the aggressor mouse in stressed mice, we calculated the SI score. The SI score of mice after CSDS was significantly lower than that before CSDS exposure (*p* < 0.001, paired *t*‐test; Figure 1f).

### 3.2 Depression‐ and Anxiety‐Like Behaviors Induced by CSDS

**FIGURE 2:**
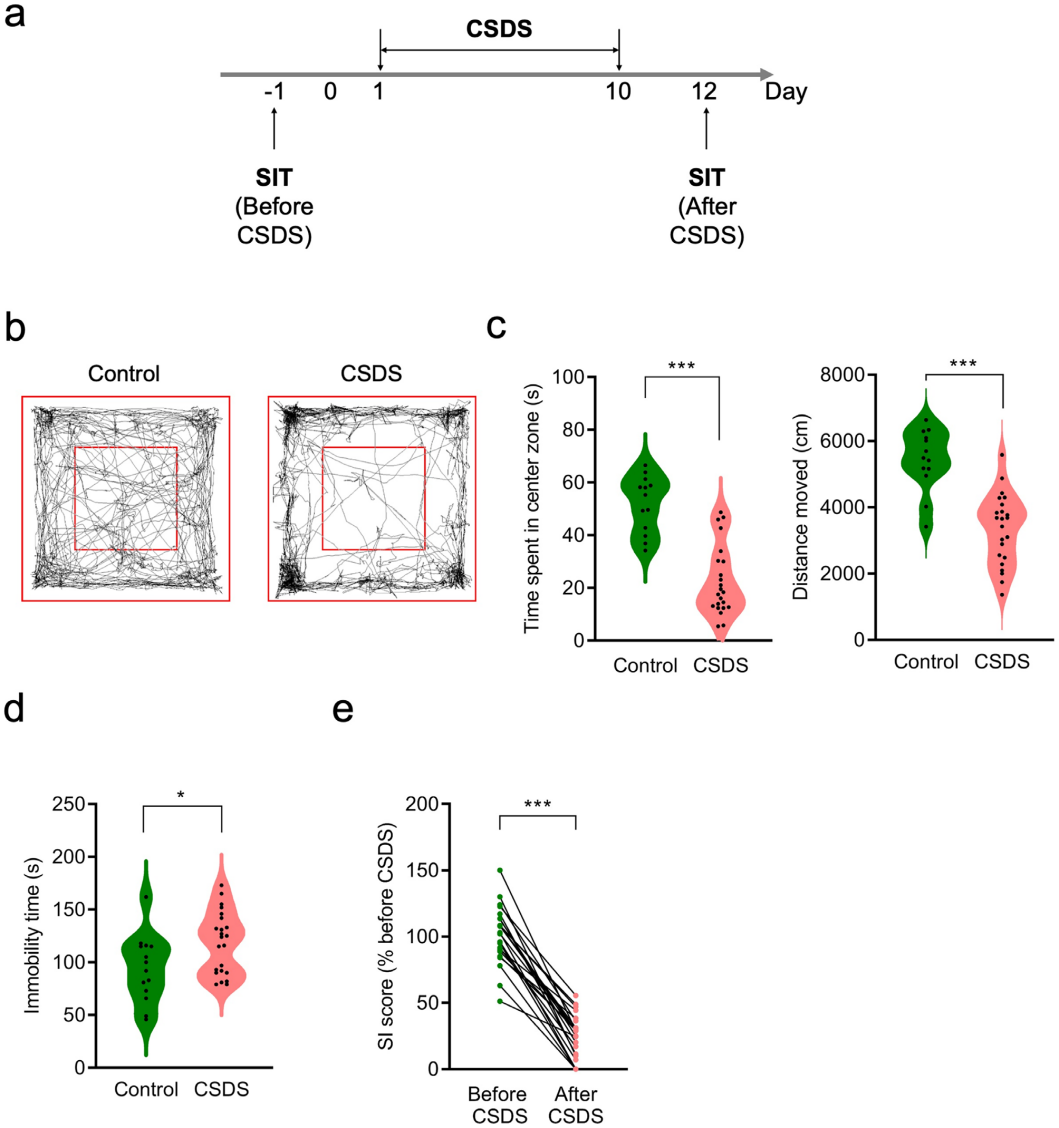
Anxiety and depression‐like behaviors induced by CSDS. (a) Experimental timeline of CSDS and subsequent depression‐related behavioral tests. Stressed (CSDS, *n* = 23) or non‐stressed (Control, *n* = 13) mice were assessed using the open‐field test (OFT), tail suspension test (TST), and SIT to evaluate depression‐related behaviors. (b) Representative tracking images of Control (left) and CSDS (right) mice in the OFT. (c) Violin plots showing the mean time in the center zone (left) and total distance traveled (right) by control and CSDS mice in the OFT. ***p < 0.001, unpaired Student's *t*‐test. (d) Violin plot showing the mean immobility time of control and CSDS mice in the TST. *p < 0.05, unpaired Student's *t*‐test. (e) SI score changes before and after CSDS in mice that underwent OFT and TST. SI score was normalized to the average score before CSDS. ***p < 0.001, paired Student's *t*‐test.

Given that CSDS‐induced reductions in social behaviors are frequently linked to depressive and anxiety‐like behaviors, we examined these behaviors in mice following CSDS exposure in this study (Figure 2a). In the OFT, stressed mice spent significantly less time and traveled a shorter total distance in the center zone compared to control mice (time spent in the center zone, total distance: *p* < 0.001, unpaired *t*‐test; Figure 2b,c). Additionally, in the TST, immobility time was significantly increased in stressed mice relative to control mice (*p* < 0.05, unpaired *t*‐test; Figure 2d). The SI score of stressed mice exhibiting anxiety‐ and depression‐like behaviors was also significantly reduced (*p* < 0.001, paired *t*‐test; Figure 2e). These findings suggest that CSDS induces not only social avoidance but also depression‐ and anxiety‐like behaviors.

### 3.3 Therapeutic Effects of DOP Agonists on Social Avoidance Induced by CSDS

**FIGURE 3:**
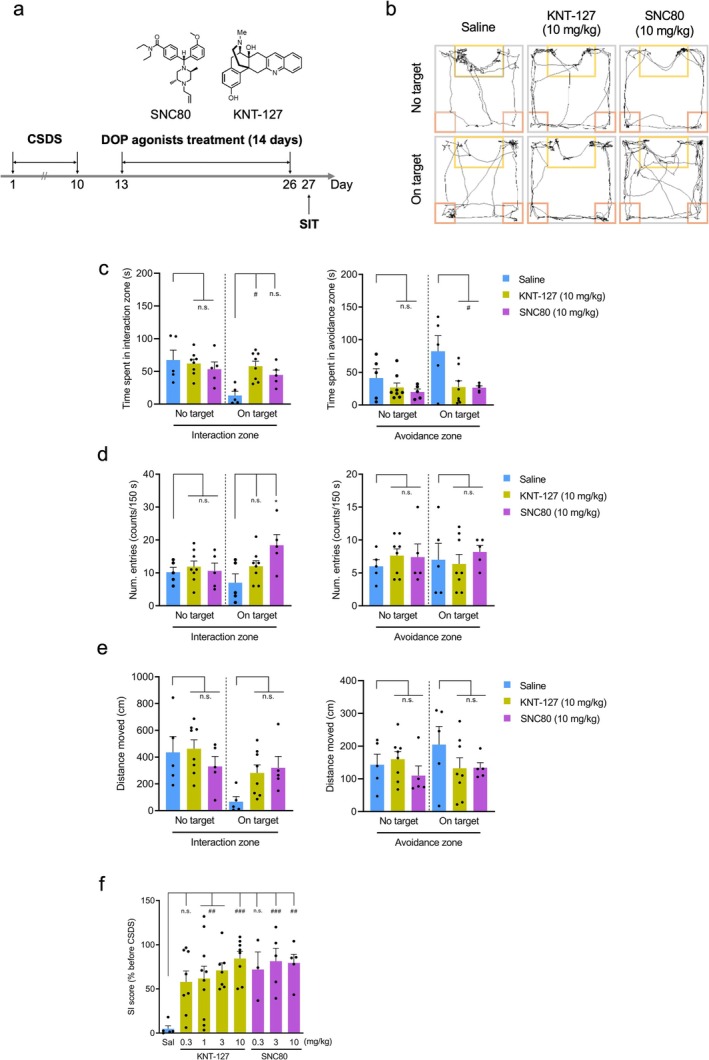
Therapeutic effects of DOP agonists on CSDS‐induced social avoidance. (a) Experimental timeline for drug administration and SIT. Following 14 days of treatment with KNT‐127 (*n* = 8), SNC80 (*n* = 5), or saline (*n* = 5), SIT was performed on stressed mice. (b) Representative tracking images of mice treated with DOP agonists (10 mg/kg) or saline during “no target” and “on target” sessions. (c) Bar graphs of mean time spent in the interaction zone (left) and avoidance zone (right) by stressed mice treated with DOP agonists (10 mg/kg) during “no target” and “on target” sessions. Data represent mean ± SEM. ^#^ *p* < 0.05, one‐way ANOVA with Tukey's test; n.s., not significant. (d) Bar graphs of mean entries into the interaction zone (left) and avoidance zone (right) by stressed mice following 14 days of DOP agonist (10 mg/kg, *n* = 5–8) or saline (*n* = 5) treatment. *p < 0.05, one‐way ANOVA with Tukey's test; n.s., not significant. (e) Bar graphs showing the mean total distance traveled in the interaction zone (left) and avoidance zone (right) by stressed mice after 14 days of DOP agonist (10 mg/kg, *n* = 5–8) or saline (*n* = 5) treatment. Data represent mean ± SEM, one‐way ANOVA with Tukey's test; n.s., not significant. (f) Dose‐dependent therapeutic effects of KNT‐127 (0.3–10 mg/kg, *n* = 7–10) and SNC80 (0.3–10 mg/kg, *n* = 3–5) on SI score. SI score was normalized to the average score before CSDS. Data represent mean ± SEM. ^##^ *p* < 0.01, ^###^ *p* < 0.001, one‐way ANOVA with Tukey's test; n.s., not significant.

We next evaluated the therapeutic effects of chronic DOP agonists administration on social behavior impairments characterized by social avoidance in mice exposed to CSDS (Figure 3a,b). A subset of mice in the CSDS model exhibits resilience to stress [12], and so DOP agonists were administered to mice showing sufficiently reduced SI scores, as shown in the histogram of SI scores in Figure S3. Based on the SI score distribution of non‐stressed control mice (ranging from 72.9 to 138.2), we classified CSDS mice with SI scores < 70 as stress‐susceptible for subsequent DOP‐agonists treatment. During the “on‐target” session, time spent in the interaction zone showed an increasing trend with SNC80 (10 mg/kg, i.p.) treatment for 14 days, and a significant increase with KNT‐127 (10 mg/kg, i.p.) treatment for 14 days compared to saline treatment (*p* < 0.05, one‐way ANOVA followed by Tukey's test; Figure 3c). However, the number of entries in the interaction zone increased significantly with SNC80 treatment, but not with KNT‐127 treatment (*p* < 0.05, one‐way ANOVA followed by Tukey's test; Figure 3d). In contrast, time spent in the avoidance zone during the “on‐target” session significantly decreased with 14‐day DOP agonist treatments compared to saline (*p* < 0.05, one‐way ANOVA followed by Tukey's test; Figure 3c). Meanwhile, there were no significant differences among the three groups in the number of entries or the total distance traveled in the avoidance zone during the “on‐target” session (*p* > 0.05, one‐way ANOVA followed by Tukey's test; Figure 3d,e).

To further explore the dose‐dependent effects of the DOP agonists, we examined their impact on SI score. Doses of KNT‐127 and KNT‐127 were selected based on previous reports [15, 21, 22, 23, 24]. While KNT‐127 at 0.3 mg/kg did not significantly increase the SI score compared to saline, doses of 1, 3, and 10 mg/kg resulted in significant increases. Similarly, SNC80 at 0.3 mg/kg did not show a significant effect on the SI score, while doses of 3 and 10 mg/kg did (KNT‐127: *p* < 0.01, *p* < 0.001; SNC80: *p* < 0.01, *p* < 0.001, one‐way ANOVA followed by Tukey's test; Figure 3f). These findings suggest that DOP agonists can mitigate CSDS‐induced social avoidance and associated depression‐like behaviors in a dose‐dependent manner.

### 3.4 Therapeutic Effects of DOP Agonists on Depression and Anxiety‐Like Behaviors Induced by CSDS

**FIGURE 4:**
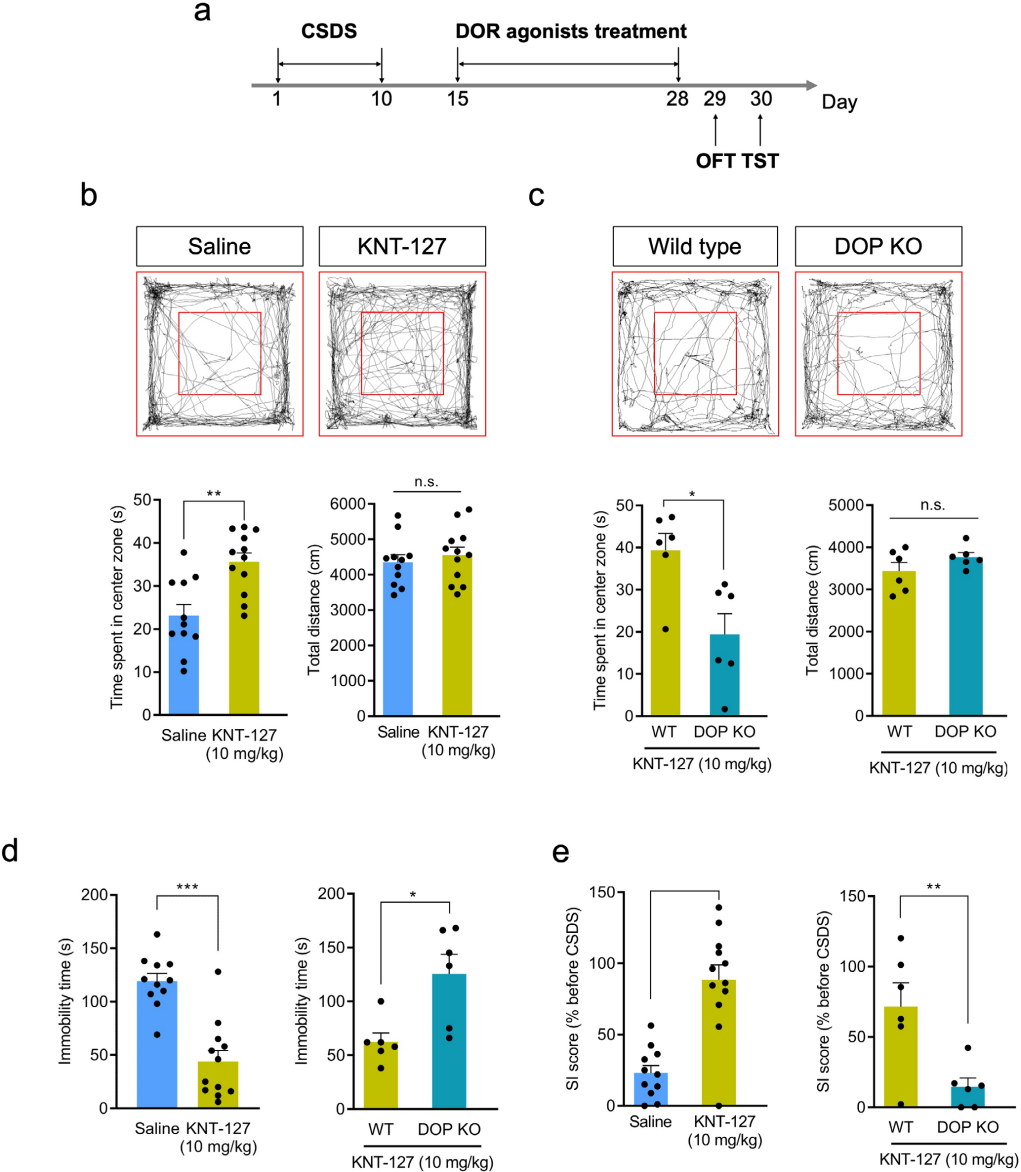
Therapeutic effects of DOP agonists on anxiety and depression‐like behaviors induced by CSDS. (a) Experimental timeline of drug treatment followed by OFT, TST, and SIT. Stressed mice were treated with either saline (*n* = 11) or KNT‐127 (10 mg/kg, *n* = 12) for 14 days. (b) (Above) Representative tracking images of stressed mice treated with KNT‐127 (right) or saline (left) in the OFT. (Below, Left) Bar graph of mean time spent in the center zone of the OFT by stressed WT mice treated with saline or KNT‐127. (Below, Right) Bar graph of the mean total distance traveled in the OFT by stressed mice treated with saline or KNT‐127. Data represent mean ± SEM. **p < 0.01, unpaired Student's *t*‐test. (c) (Above) Representative tracking images of stressed WT (left) or DOP KO (right) mice treated with KNT‐127 in the OFT. (Below, Left) Bar graph of the mean time spent in the center zone of the OFT by stressed WT (*n* = 6) or DOP KO (*n* = 6) mice treated with KNT‐127. (Below, Right) Bar graph of the mean total distance traveled in the OFT by stressed WT (*n* = 6) or DOP KO (*n* = 6) mice treated with KNT‐127. Data represent mean ± SEM. *p < 0.05, unpaired Student's *t*‐test. (d) (Left) Bar graph showing mean immobility time in the TST for stressed mice treated with saline (*n* = 11) or KNT‐127 (*n* = 12) for 14 days. (Right) Bar graph showing the mean immobility time in the TST by stressed WT (*n* = 6) or DOP KO (*n* = 6) mice treated with KNT‐127 for 14 days. Data represent mean ± SEM. *p < 0.05, ***p < 0.001, unpaired Student's *t*‐test. (e) (Left) Bar graph showing mean SI score of stressed mice treated with saline (*n* = 11) or KNT‐127 (*n* = 12) after performing OFT and TST. (Right) Bar graph showing the mean SI score of CSDS‐WT (*n* = 6) or DOP KO (*n* = 6) mice after performing OFT and TST. **p < 0.01, ***p < 0.001, unpaired Student's *t*‐test.

We next investigated whether chronic administration of DOP agonists (10 mg/kg) over 14 days alleviates depressive and anxiety‐like behaviors induced by CSDS (Figure 4a). In the OFT, KNT‐127 (10 mg/kg, i.p.) significantly increased the time spent in the center zone in CSDS‐WT mice (*p* < 0.01, unpaired *t*‐test; Figure 4b, left), without affecting the total distance traveled (*p* > 0.05, unpaired *t*‐test; Figure 4b, right). KNT‐127 was administered to DOP KO mice, which exhibit anxiety‐ and depression‐like behaviors followed by CSDS induction to investigate whether this effect was due to DOP activation (Figure S4). Consequently, such KNT‐127‐induced anxiolytic effects in CSDS‐WT mice were abolished in DOP KO mice (*p* < 0.05, unpaired *t*‐test; Figure 4c). In the TST, KNT‐127 markedly reduced immobility time in CSDS‐WT mice, which is associated with depression‐like behaviors (*p* < 0.001, unpaired *t*‐test; Figure 4d, left), and the antidepressant effect was not observed in DOP KO mice (*p* < 0.05, unpaired *t*‐test; Figure 4d, right). Additionally, in the SIT, the SI score significantly increased in mice showing improvements in depressive and anxiety‐like behaviors (*p* < 0.001, unpaired *t*‐test; Figure 4e, left), but no effect was observed in DOP KO mice (*p* < 0.01, unpaired *t*‐test; Figure 4e, right). These findings suggest that mice exhibiting reduced social avoidance due to DOP agonists show improvements in general depressive and anxiety‐like behaviors via DOP.

### 3.5 Comparison of the Therapeutic Effects of DOP Agonists and SSRI on Social Avoidance Induced by CSDS

**FIGURE 5:**
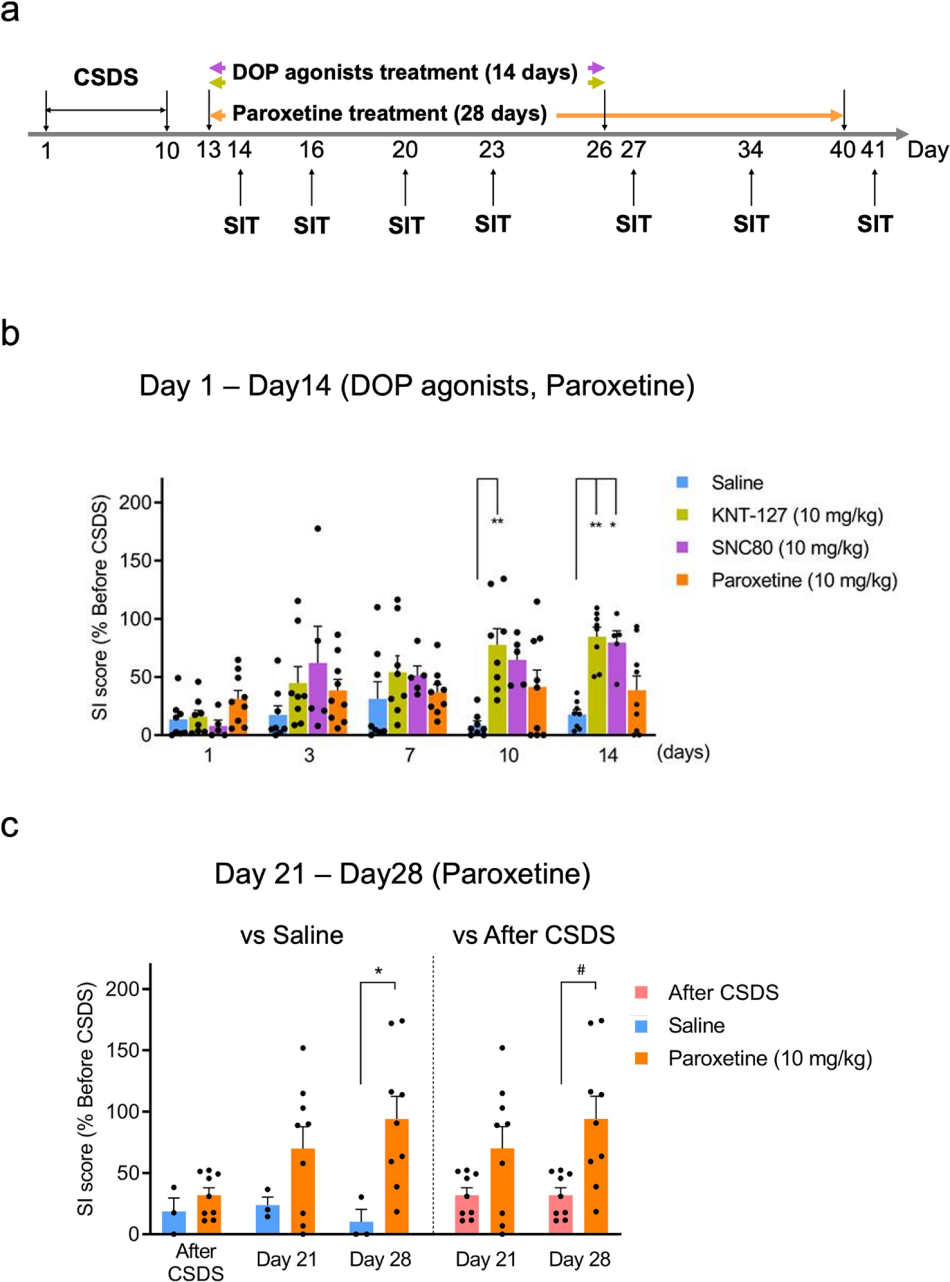
Comparison of therapeutic effects of DOP agonists and SSRI on CSDS‐induced social avoidance. (a) Experimental timeline of DOP agonists and paroxetine treatment and SIT. Mice were treated with KNT‐127 (10 mg/kg, *n* = 8), SNC80 (10 mg/kg, *n* = 5), or paroxetine (10 mg/kg, *n* = 9) for 14 and 28 days, respectively. SIT was conducted 18 h after administration on Days 1, 3, 7, 10, 14, 21, and 28. (b) Time‐course changes in the therapeutic effects of DOP agonists and paroxetine during the first 14 days of treatment. Data represent the mean ± SEM. *p < 0.05, **p < 0.01, one‐way ANOVA followed by Tukey's test. (c) Delayed therapeutic effects of paroxetine assessed on Days 21 and 28. (Left) comparison of SI scores between saline‐ and paroxetine‐treated CSDS mice. (Right) comparison between SI scores measured after CSDS and those following paroxetine treatment on Days 21 and 28. Data represent the mean ± SEM. (left) *p < 0.05 versus saline‐treatment group, one‐way ANOVA followed by Tukey's test. (right) ^#^ *p* < 0.05 vs. After‐CSDS SI score, paired Student's *t*‐test.

To assess the comparative therapeutic effects of DOP agonists versus the SSRI paroxetine, we chronically administered KNT‐127 and SNC80 (10 mg/kg, i.p., Figure 3f) to stressed mice over a 14‐day period, with SIT conducted approximately 18 h after administration on Days 1, 3, 7, 10, and 14. Paroxetine (10 mg/kg, i.p.) [25, 26] was also administered chronically for 28 days, with SIT conducted approximately 18 h after administration on Days 1, 3, 7, 10, 14, 21, and 28 (Figure 5a).

Results indicated that KNT‐127 and SNC80 produced significant improvements in the SI score by Days 10 and 14 of administration, respectively, and that paroxetine showed no such effect within the same timeframe (KNT‐127: *p* < 0.01; SNC80: *p* < 0.05; paroxetine: *p* > 0.05 vs. saline group, one‐way ANOVA followed by Tukey's test; Figure 5b). In contrast, continued administration of paroxetine led to a tendency toward improvement on Day 21 and a significant increase in the SI score by Day 28, compared to both the saline‐treated group and the After‐CSDS SI score (*p* < 0.05 vs. saline group, one‐way ANOVA followed by Tukey's test; *p* < 0.05 vs. After‐CSDS SI score, paired *t*‐test; Figure 5c). These findings suggest that DOP agonists may offer more rapid and effective therapeutic benefits than traditional SSRIs like paroxetine.

## 4 Discussion

Animal models are essential in preclinical research for assessing the therapeutic potential of novel compounds, especially for psychiatric disorders like depression, where social and environmental factors play a critical role. Validity in these models is determined by three key criteria: face validity (the extent to which the model replicates human symptoms), construct validity (support for the underlying theoretical basis), and predictive validity (the model's accuracy in forecasting clinical therapeutic outcomes) [13]. However, traditional models, such as unpredictable stress and restraint stress [27, 28, 29], often fail to capture the full complexity of psychiatric symptoms, which involve higher cognitive functions like perception, memory, and emotion—factors that are challenging to measure in animals. While useful for detecting the acute effects of antidepressants, these models are limited in their capacity to assess long‐term therapeutic outcomes [30], an essential consideration in treating chronic depression. Therefore, developing more sophisticated models is crucial for accurately representing the chronic nature of psychiatric disorders.

The olfactory bulbectomy (OBX) model exhibits high face validity by replicating behavioral, neurochemical, and neuroendocrine changes similar to those observed in clinical depression [31]. This model has been employed to investigate the early onset of DOP agonist effects [32]. However, despite its usefulness, the OBX model has limited construct validity, as it does not directly correspond to a specific pathology in human depression. To address these limitations, we used the more clinically relevant CSDS model in the present study.

The CSDS model is well‐established as a reliable tool in depression research, meeting crucial validity criteria—face, construct, and predictive. This model induces behavioral alterations, such as reduced social interaction, accompanied by anhedonia and decreased locomotor activity. Neuroendocrine changes, including HPA axis activation and elevated blood corticosterone levels, have been documented [33], along with impairments in thermoregulation [34], immune function, and autonomic circadian rhythms [35]. Consistent with the broader understanding of depression, which often includes symptoms like anxiety and irritability [2], our study observed similar anxiety‐like behaviors in CSDS mice [36]. These physiological and behavioral responses closely mirror clinical depression symptoms, further supporting the high face validity of the CSDS model. Notably, the model demonstrates high construct validity by replicating clinical pathology through repeated social defeat, leading to depression‐like behaviors. In our study, chronic paroxetine treatment over 3–4 weeks was necessary to produce therapeutic effects, further underscoring the high predictive validity of the CSDS model. Given that the CSDS model effectively reflects stress patterns prevalent in modern society, our findings on DOP agonists in this model—demonstrating faster and more favorable therapeutic outcomes than current antidepressants—hold significant clinical relevance.

In the present study, DOP agonists alleviated CSDS‐induced social avoidance toward an aggressor mouse more rapidly than paroxetine. The mechanisms underlying depression are complex and involve multiple factors, with chronic stress shown to significantly impact neurogenesis, particularly in the dentate gyrus (DG) of the hippocampus [37, 38, 39]. Chronic stress impairs the proliferation of neural progenitor cells, thereby inhibiting neurogenesis. This effect is evidenced by a reduction in BrdU‐positive cells [40, 41, 42, 43] and decreased expression of markers specific to neural stem and progenitor cells [40, 44]. The inhibition of neurogenesis due to chronic stress is considered a key contributor to the development of depression. However, chronic SSRI treatment has been shown to increase neurogenesis and improve anxiety‐like behaviors [45, 46, 47, 48, 49]. Mature granule cells in the DG reportedly shift into a “dematured” state following chronic SSRI treatment, suggesting that lost cellular functions may be restored [37]. This neurogenesis and dematuration are mediated by 5‐HT_1_ and 5‐HT_4_ receptors [37], indicating that elevated 5‐HT levels through chronic SSRI treatment may promote transcriptional changes via dematuration of granule cells, thereby enhancing neurogenesis and contributing to antidepressant effects [38, 39, 50]. However, SSRIs typically require time to maintain sufficient 5‐HT levels in the synaptic cleft, resulting in a slower increase in neurogenesis.

In contrast, the molecular basis underlying DOP agonists' antidepressant effects is less understood. Recent research suggests that KNT‐127 increases extracellular glutamate levels by activating DOP in the medial prefrontal cortex [51, 52]. This promotion of glutamate release by KNT‐127 parallels the antidepressant mechanism of ketamine [53, 54], which may account for KNT‐127's rapid therapeutic effects, distinguishing it from SSRIs [55, 56].

DOP agonists can modulate glutamatergic neurotransmission and the monoaminergic system, which is closely involved in emotional processing. For instance, KNT‐127 increases dopamine release in the striatum and medial prefrontal cortex [51], while DADLE, DPDPE, and deltrophin II elevate extracellular serotonin levels in the dorsal raphe nucleus [57]. Based on these findings, the DOP system may regulate emotional function by controlling the activity of the monoaminergic system upstream. Therefore, dysfunction in the DOP system can significantly impact emotional regulation. In mice and rats exposed to various stressors, mRNA and protein expression levels of DOP and enkephalin are reduced [23, 58, 59, 60, 61, 62, 63, 64], suggesting that stress may downregulate endogenous enkephalin‐DOP signaling. DOP agonists exert their therapeutic effects by directly binding to DOP and activating DOP signaling. This process may underlie the more rapid therapeutic effects of DOP agonists compared to paroxetine.

In this study, KNT‐127 exhibited significant therapeutic effects after 10 days of repeated treatment, while SNC80 showed significant effects after 14 days. Previously, we demonstrated that DOP agonists have been reported to reduce PTSD‐like anxiety behaviors induced by contextual fear conditioning [65, 66]. Interestingly, recent studies indicate that KNT‐127, but not SNC80, more effectively facilitates the extinction of contextual fear memories [65], which may explain its more rapid improvement of PTSD‐like symptoms. Similar to fear conditioning, social avoidance induced by CSDS—accompanied by depression and anxiety—has a component of “contextual conditioning.” [67] This conditioning implies that stressed mice associate the chamber used for the SIT with prior fear or stress from CSDS, and their fear response reactivates upon re‐exposure. In this study, a single administration of DOP agonists did not significantly improve social avoidance triggered by visual recognition of an aggressor mouse in CSDS. However, chronic administration led to a marked improvement in social avoidance and depressive behaviors. This suggests that repeated administration of DOP agonists may help “overwrite” the fear memory associated with CSDS, establishing “new contextual conditioning” through repeated anxiolytic effects. The rapid therapeutic onset observed in KNT‐127 compared to SNC80 may stem from its ability to facilitate fear memory extinction. This extinction effect is also noted with ketamine [68], suggesting that compounds capable of both antidepressant and fear memory extinction effects may hold promise as rapid‐acting antidepressants.

In conclusion, our study demonstrates that DOP agonists effectively reduce CSDS‐induced social avoidance behaviors, as well as depression‐ and anxiety‐like behaviors, in a model that closely mimics clinical depression. Additionally, we found that DOP agonists provide the extinction of contextual fear memory more rapidly than existing antidepressants, which are often associated with delayed action and substantial side effects. Generally, traditional DOP agonists such as SNC80 show convulsion at therapeutic doses in mice, but KNT‐127 shows no convulsive effects even at high doses [69]. Given these limitations in conventional treatments, DOP agonists, especially KNT‐127 and its derivatives, show significant potential as novel antidepressants, offering faster efficacy and potentially fewer side effects.

## Author Contributions

A.T., Y.N., T.S., and H.N. designed the study. A.T., Y.N., and K.S. conducted the experiments and analyzed the data. K.K. and K.I. synthesized and provided KNT‐127. A.T., Y.N., and S.M. provided transgenic mice. A.T., Y.N., A.S., T.S., and H.N. wrote the manuscript. All authors discussed the results and the manuscript.

## Ethics Statement

All animal experiments were approved by the Institutional Animal Care and Use Committee of the University of Tsukuba, and all procedures were conducted in accordance with the University's Guidelines for Animal Experiments. The animal studies were reviewed and approved by the Institutional Animal Care and Use Committee of the University of Tsukuba (approval no. 18‐178, 19‐309, 20‐287, 20‐289, 21‐313, 21‐321, 22‐355, 23‐282, 24‐061).

## Consent

The authors have nothing to report.

## Conflicts of Interest

The authors declare no conflicts of interest.

## Supporting information


**Figure S1:** Generation of Oprd1‐*Cre* knock‐in mice by CRISPR Cas9 system.
**Figure S2:** Two clusters of CSDS mice can be identified on the basis of the time spent in the avoidance/interaction zone in the SIT, both before and after CSDS (related to Figure 1).
**Figure S3:** Histogram showing the distribution of SI scores in CSDS mice and control mice (related to Figures 1 and 5).
**Figure S4:** Social avoidance, anxiety, or depression‐like behaviors induced by CSDS in WT or DOP KO mice (related to Figure 4).

## Data Availability

The raw data of this study are available in the Supporting Information. Owing to a prior laboratory computer hardware failure, a subset of the raw tracking‐image data could not be submitted.
