# Supplementary material for: Rapid‐Onset Therapeutic Effects of Delta Opioid Receptor Agonists on Depression‐Like Behaviors Induced by Chronic Social Defeat Stress
Source: Neuropsychopharmacol Rep. 2025 Sep 29;45(4):e70059. doi: 10.1002/npr2.70059 (PMC12479375; doi:10.1002/npr2.70059)

KNT-127_10mg (Before CSDS)
(related to Figure 1)

(Left: No target
Right: On target)

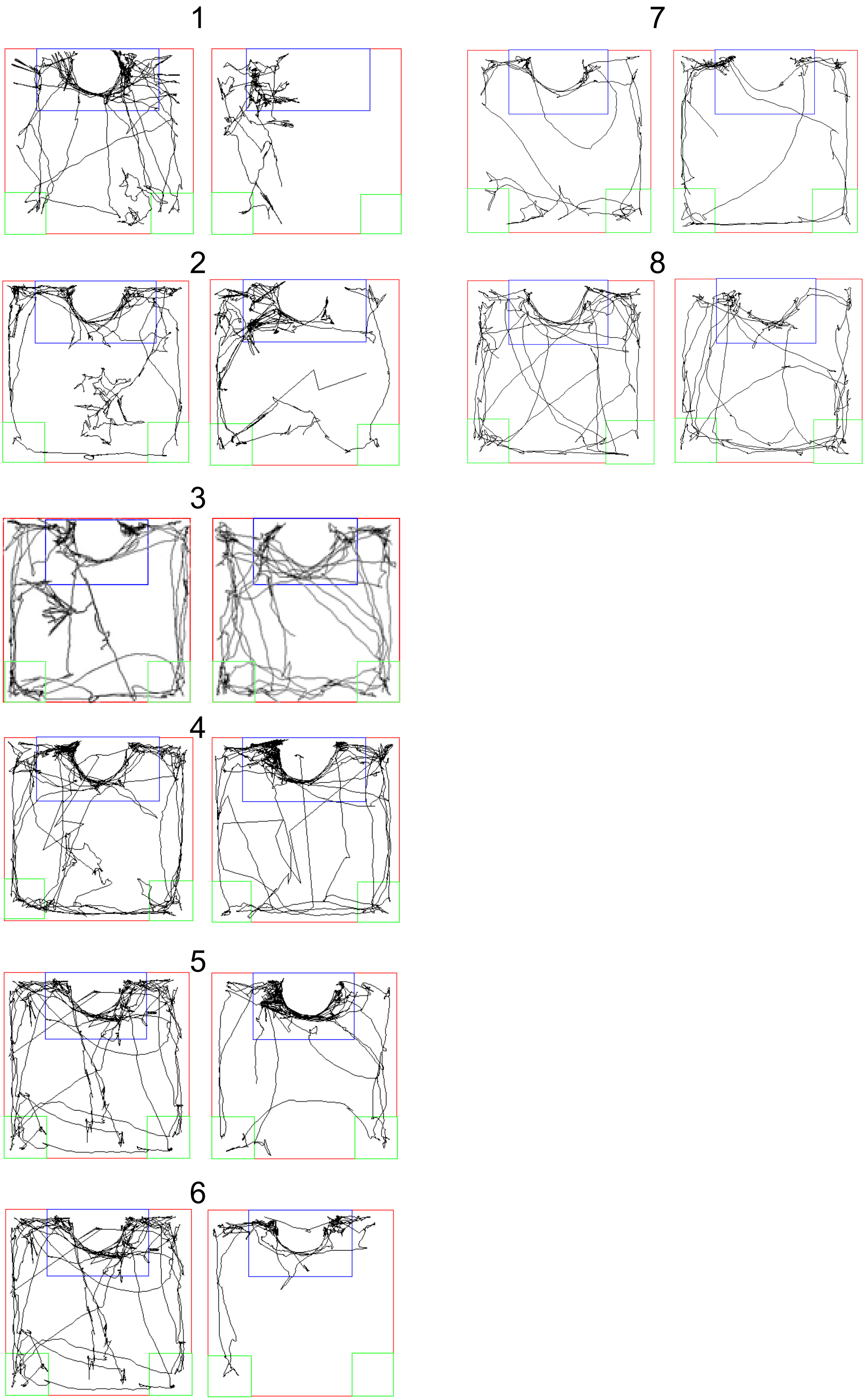

KNT-127_10mg (After CSDS)
(related to Figure 1)

(Left: No target
Right: On target)

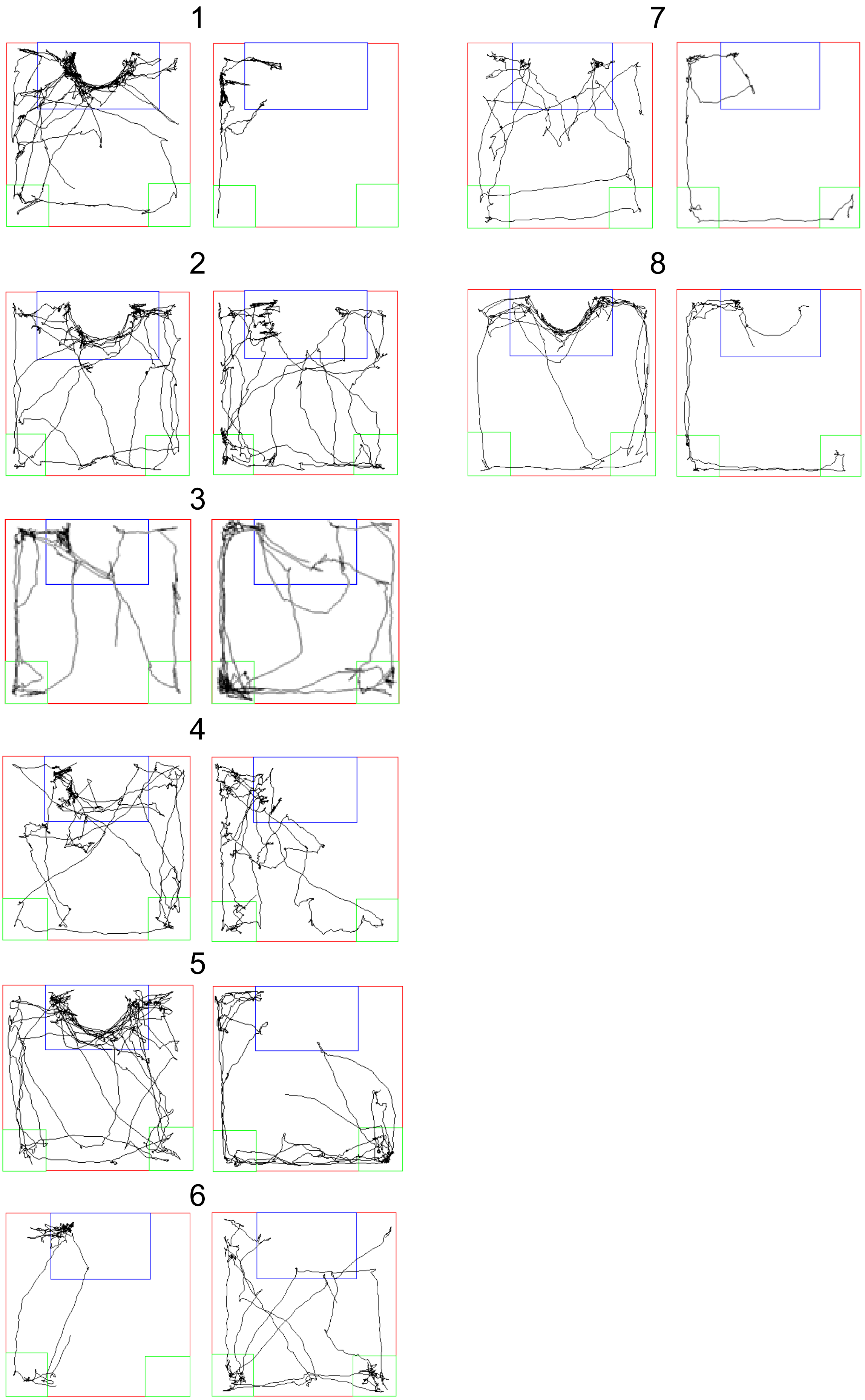

KNT-127_10mg (Day 1)
(related to Figure 5)

(Left: No target
Right: On target)

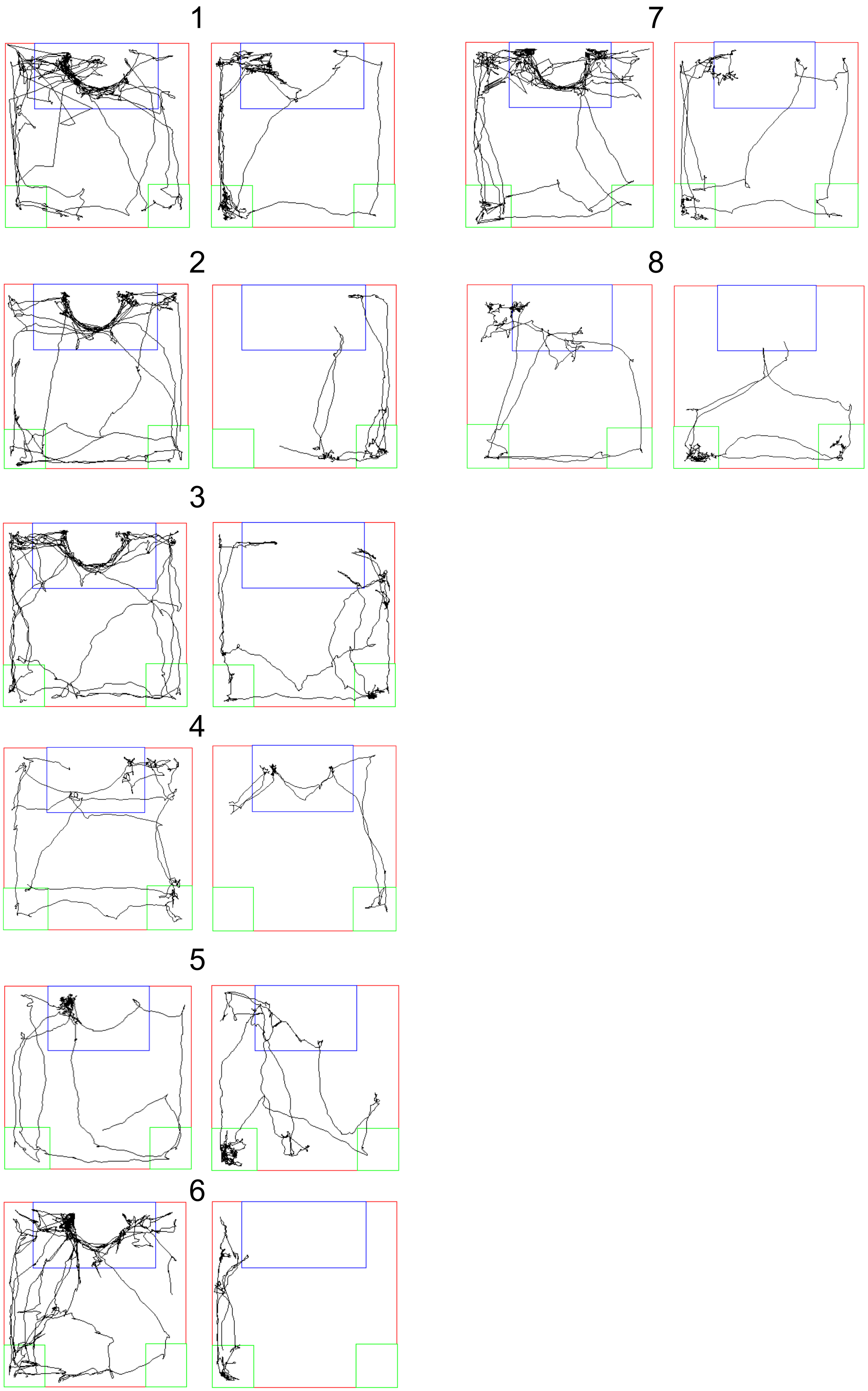

(Left: No target
Right: On target)

KNT-127_10mg (Day 3)
(related to Figure 5)

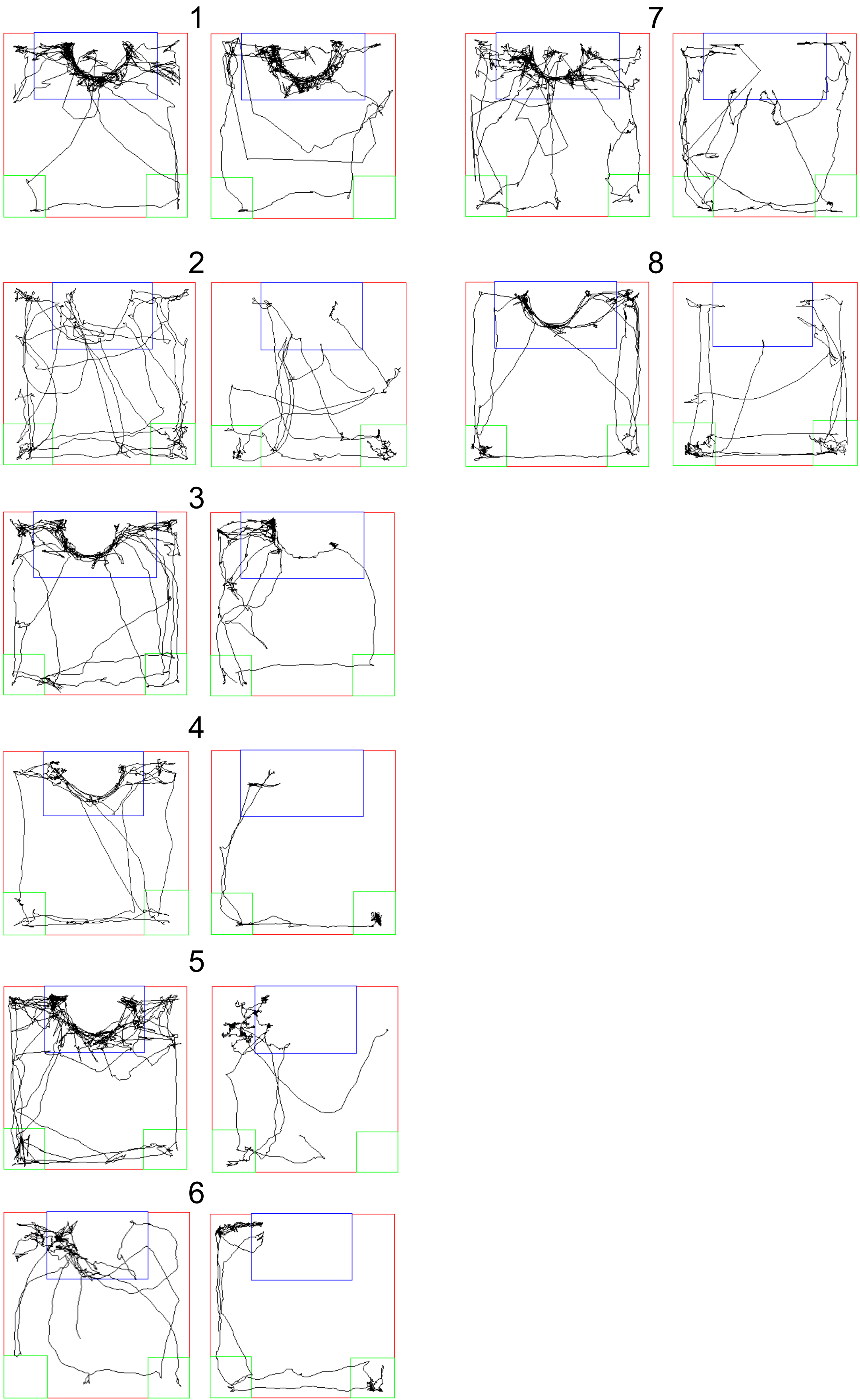

(Left: No target
Right: On target)

KNT-127_10mg (Day 7)
(related to Figure 5)

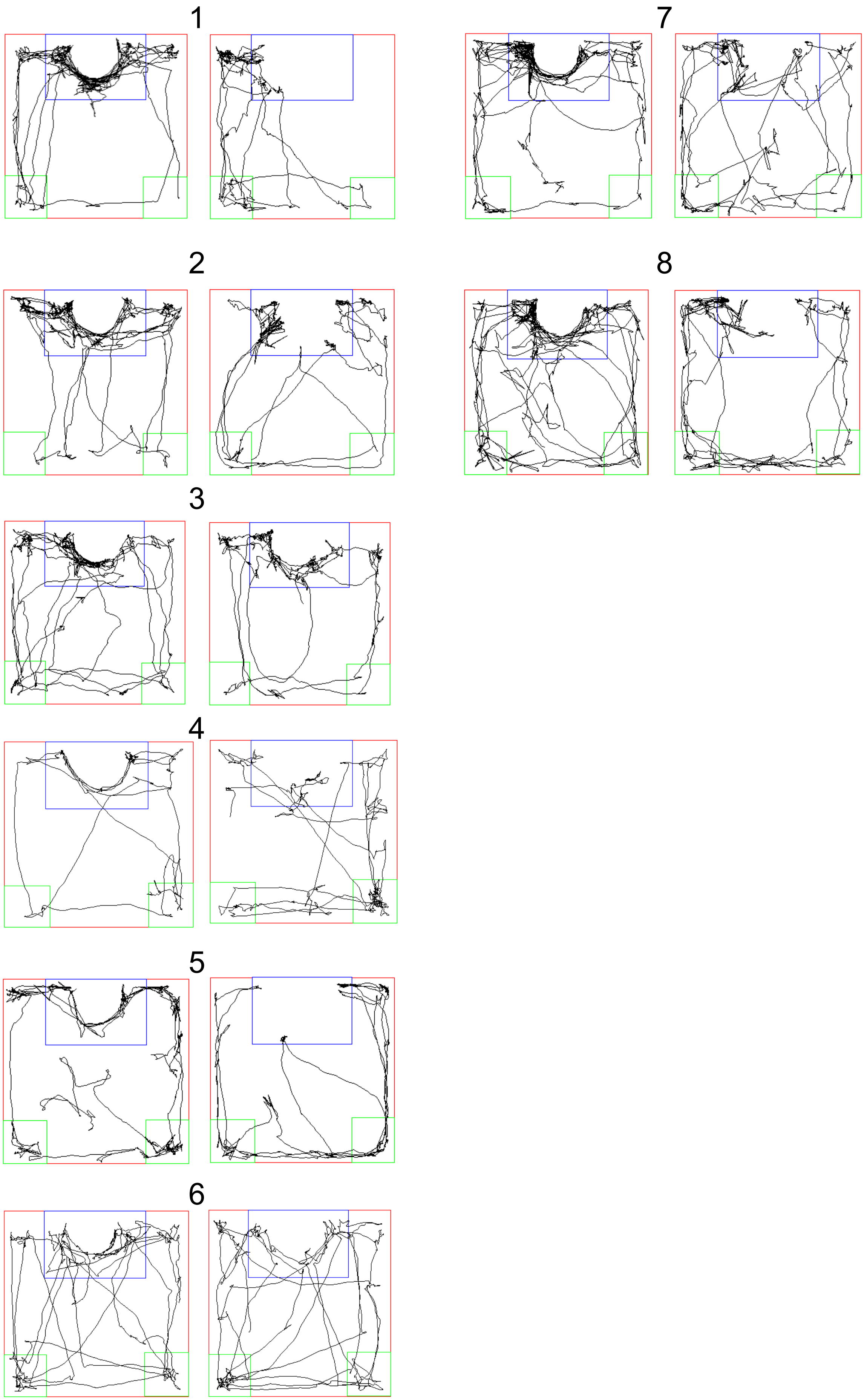

(Left: No target
Right: On target)

KNT-127_10mg (Day 10)
(related to Figure 5)

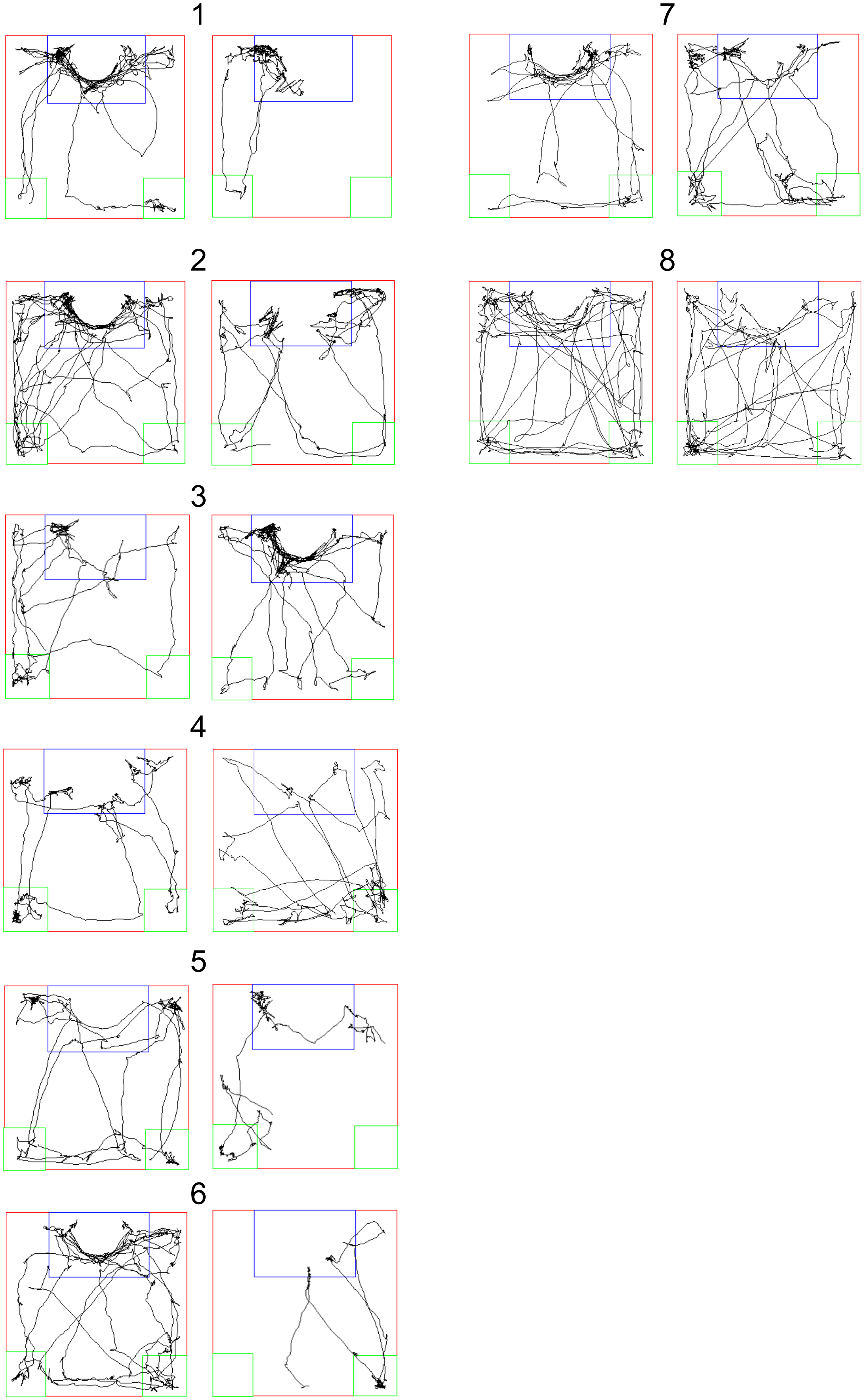

KNT-127_10mg (Day 14)
(related to Figure 3, 5)

(Left: No target
Right: On target)

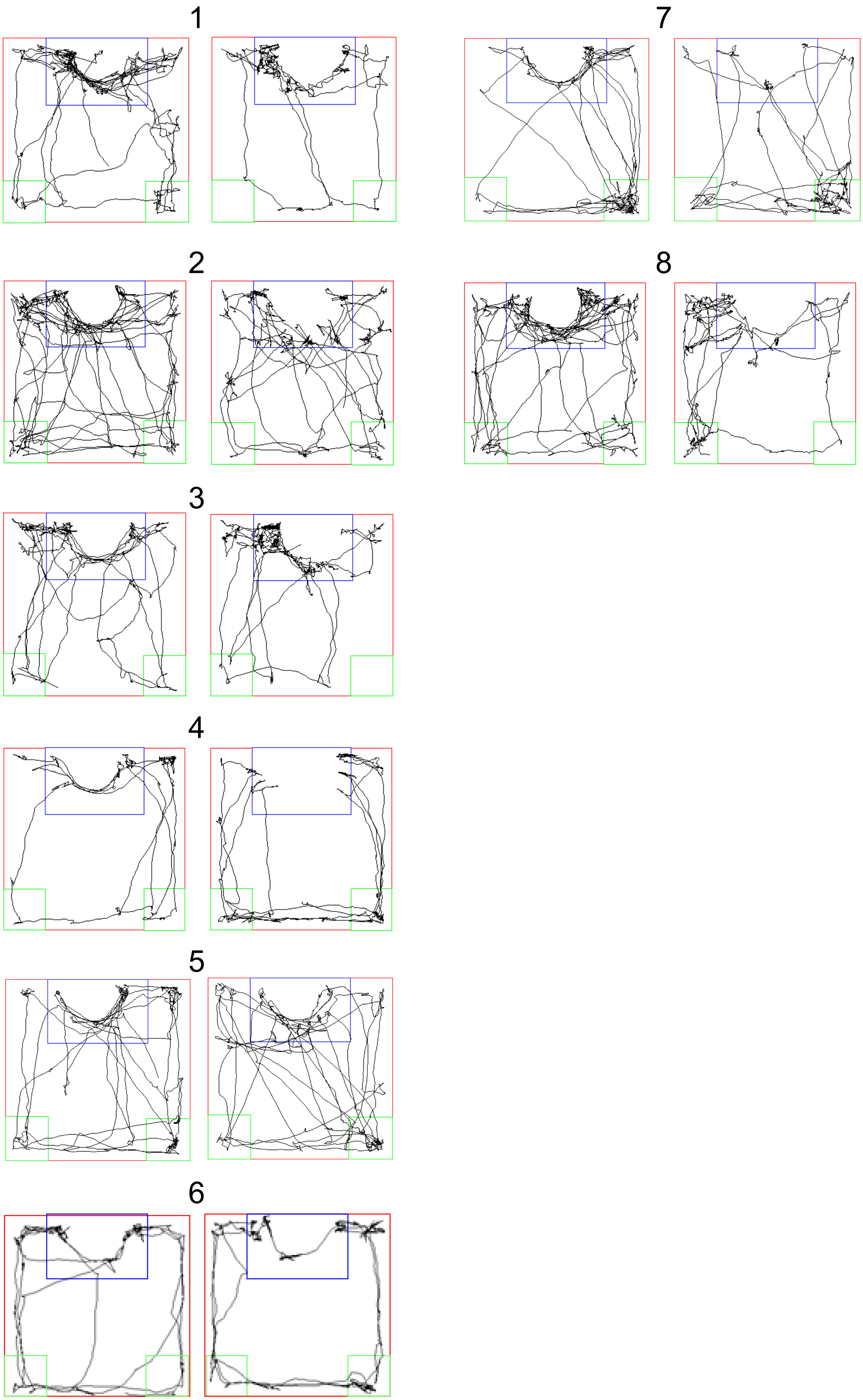

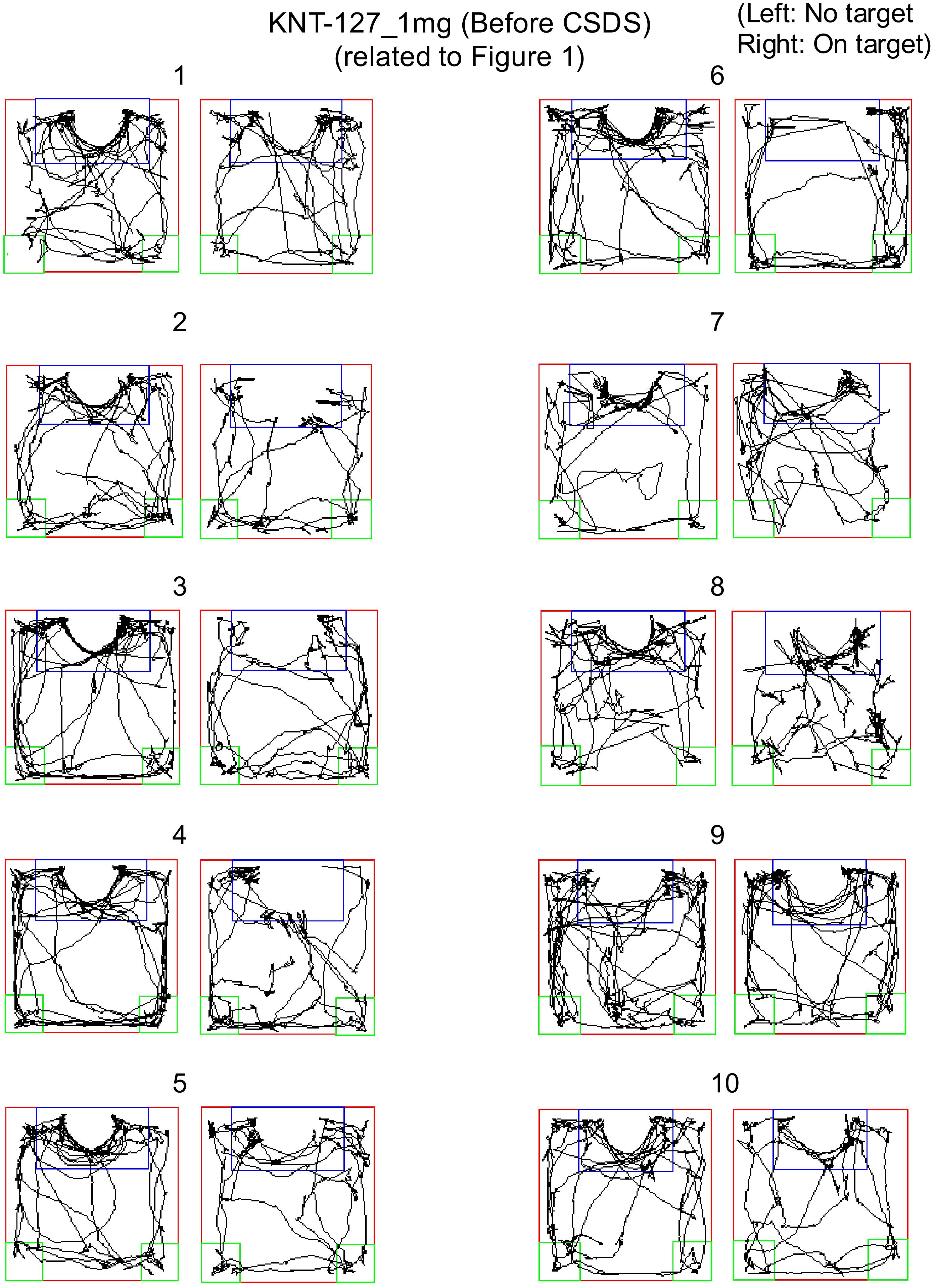
KNT-127_1mg (Before CSDS)
(related to Figure 1)
(Left: No target
Right: On target)
1
2
3
4
5
6
7
8
9
10

KNT-127_1mg (After CSDS)
(related to Figure 1)

(Left: No target
Right: On target)

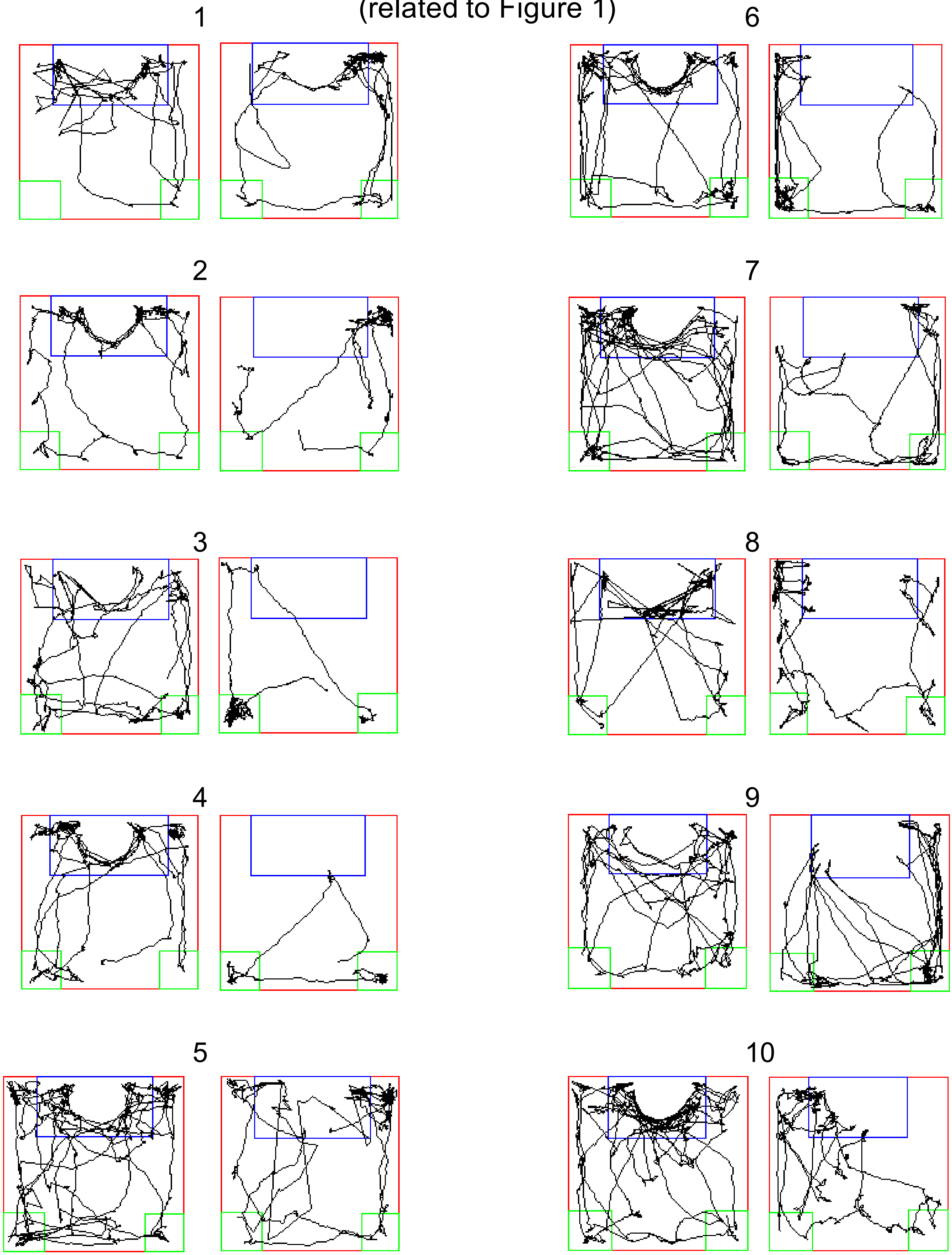

KNT-127_1mg (Day 14)
(related to Figure 3)

(Left: No target
Right: On target)

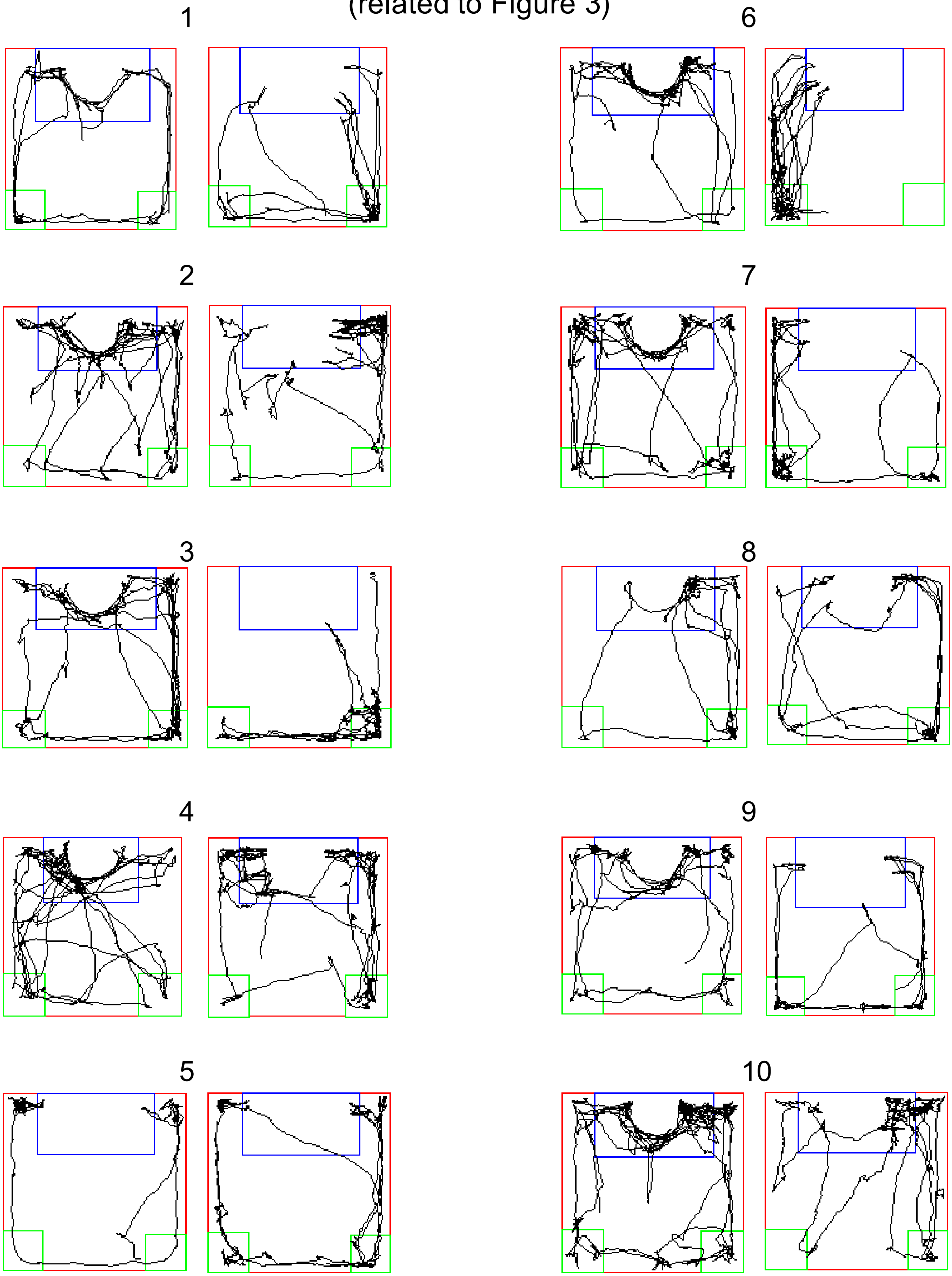

SNC80_3 mg (Before CSDS)
(related to Figure 1)

(Left: No target
Right: On target)

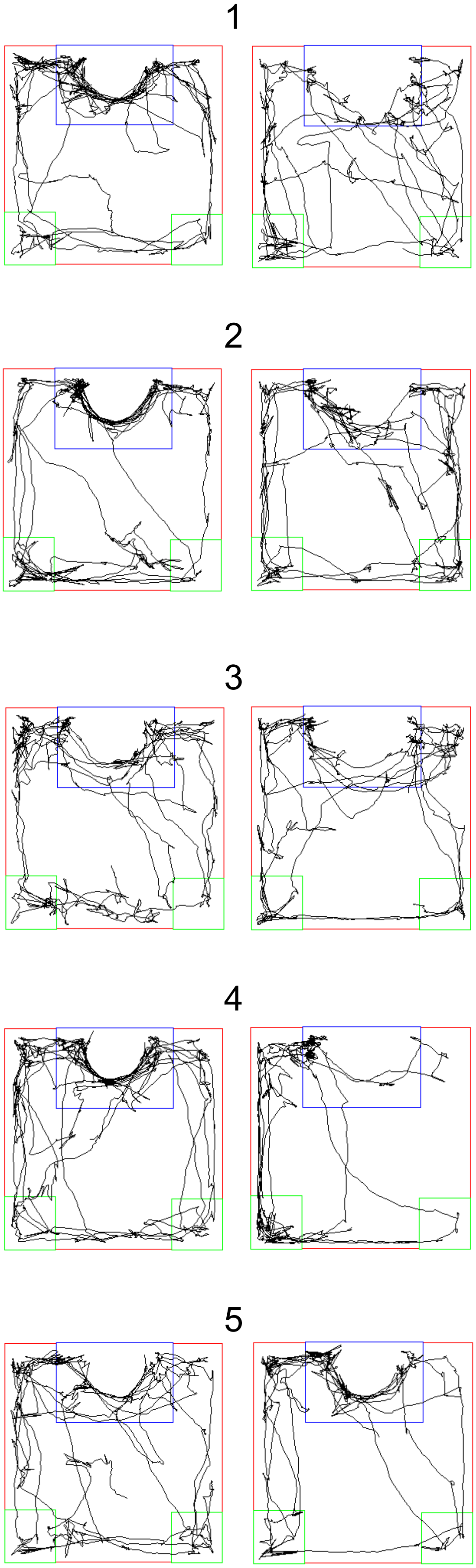

SNC80_3mg (After CSDS)
(related to Figure 1)

(Left: No target
Right: On target)

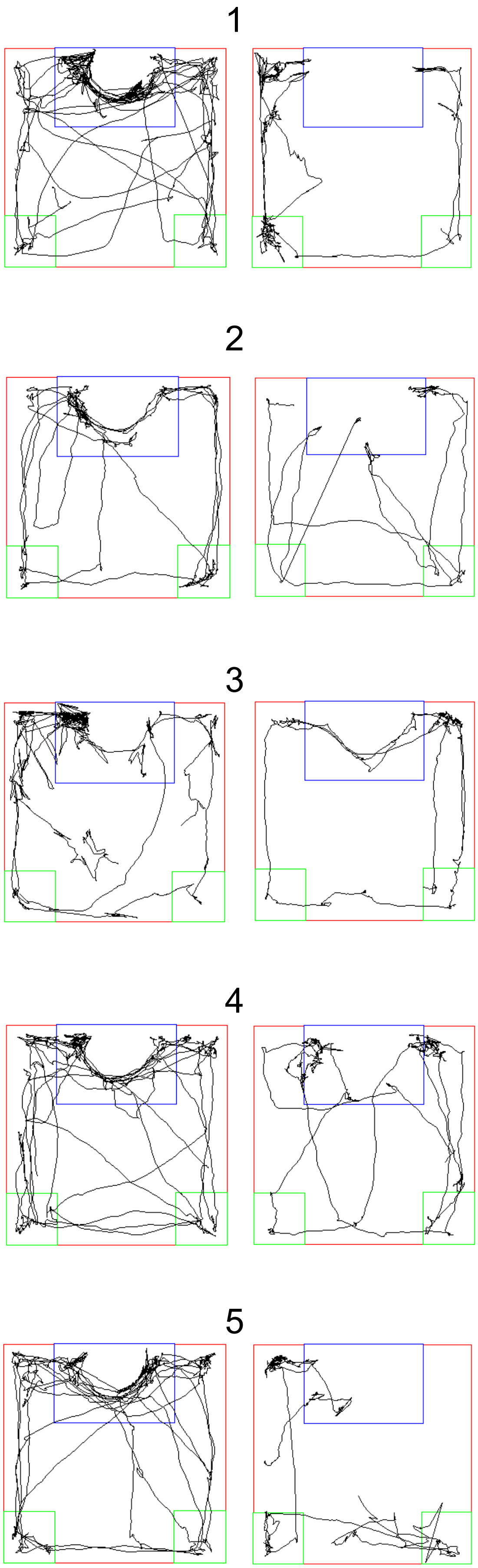

SNC80_3 mg (Day 14)
(related to Figure 3)

(Left: No target
Right: On target)

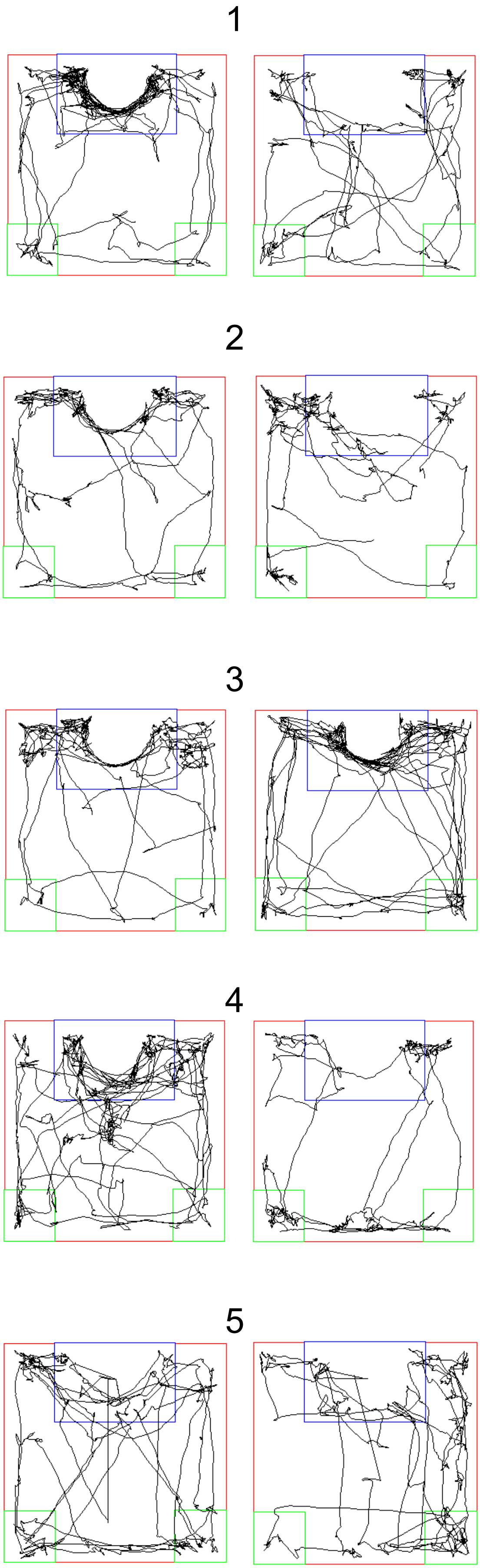

(Left: No target
Right: On target)

SNC80_10 mg (Day 14)
(related to Figure 3)

3

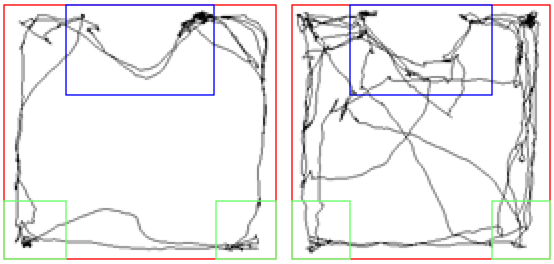

Saline (Before CSDS) (related to Figure 1)

(Left: No target
Right: On target)

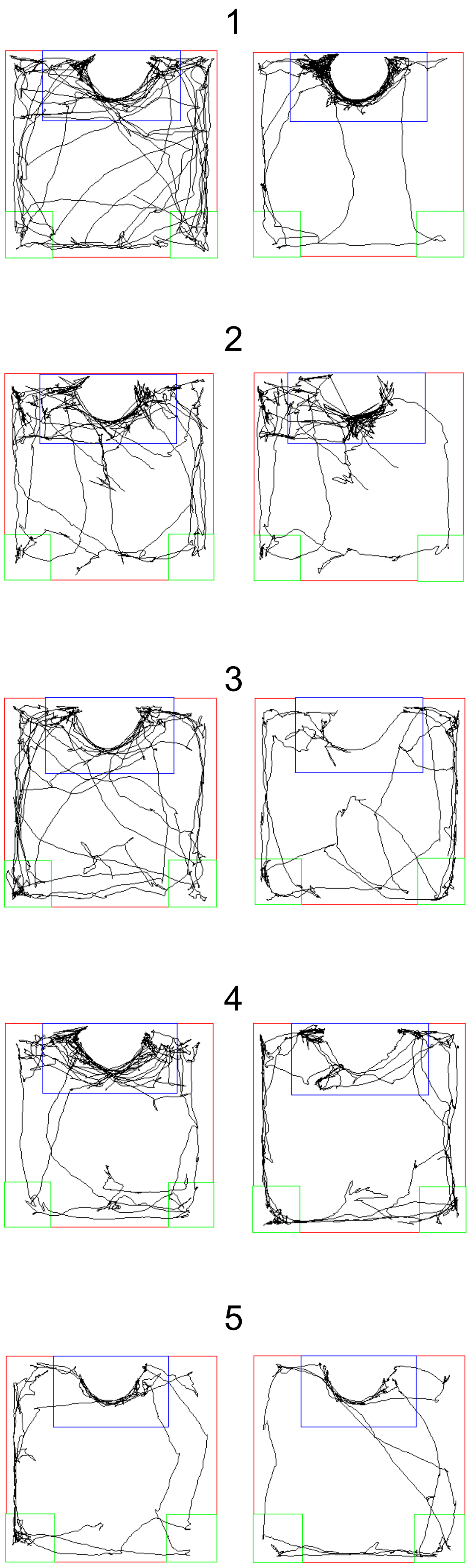

Saline (After CSDS) (related to Figure 1)

(Left: No target
Right: On target)

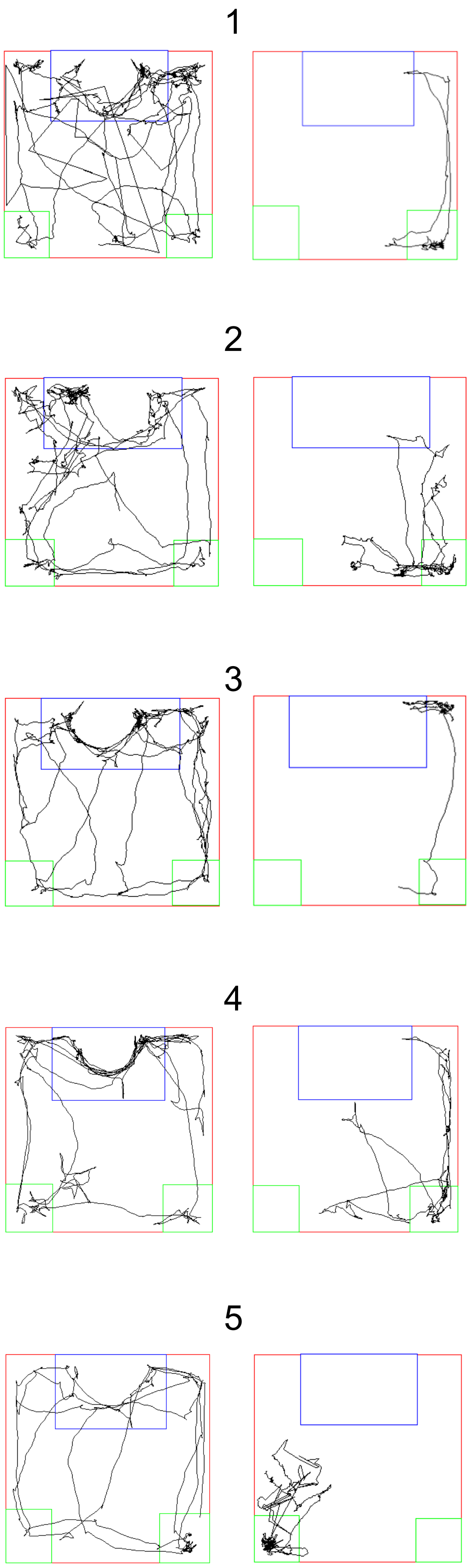

Saline (Day 14) (related to Figure 3, 5)

(Left: No target
Right: On target)

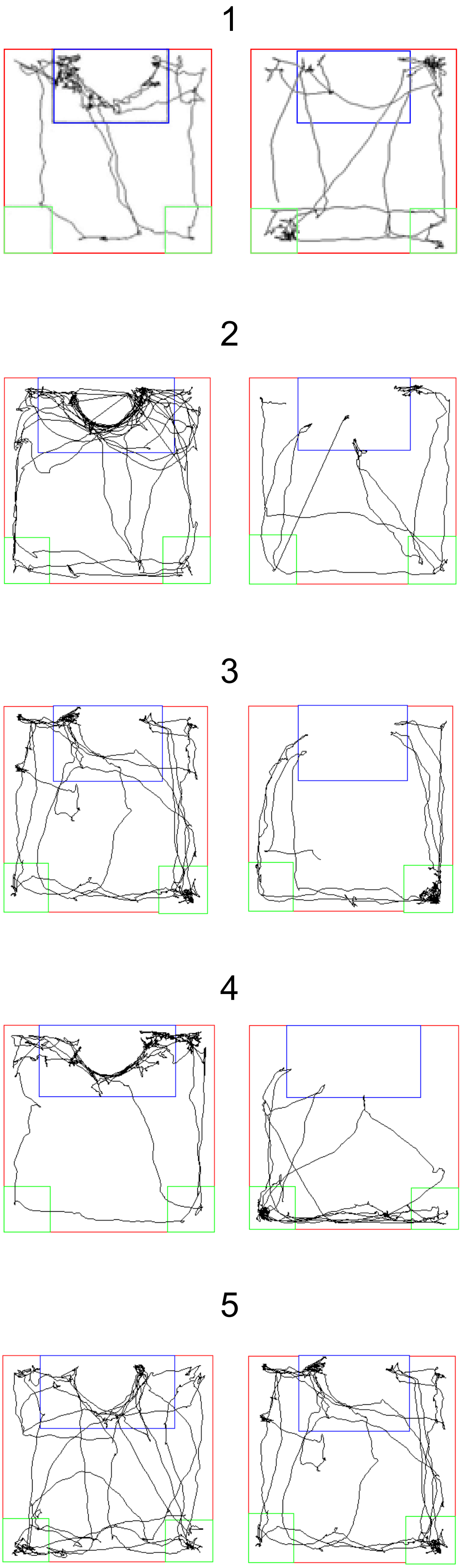

WT-CSDS After CSDS (related to Figure 2)

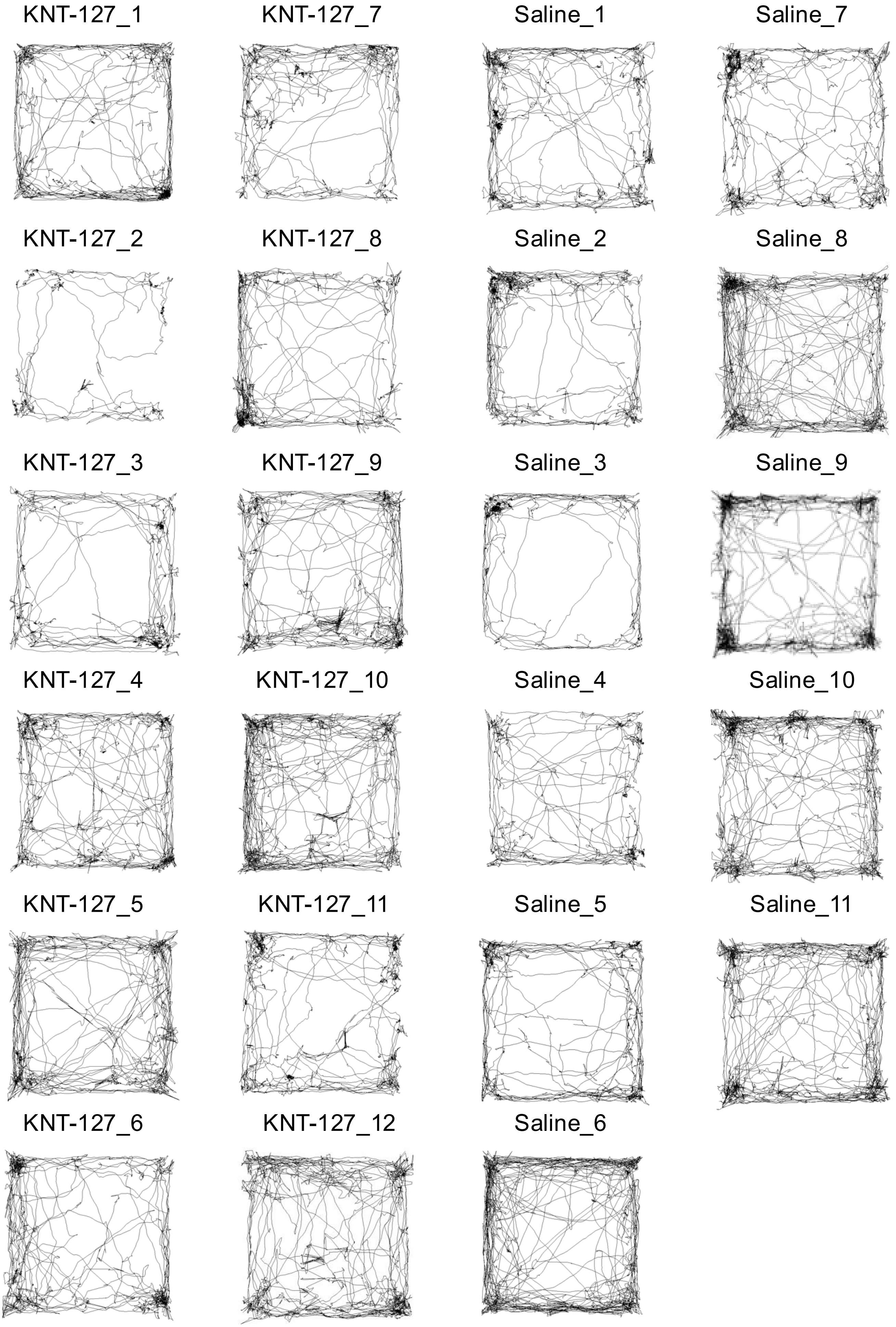

WT-Control After CSDS (related to Figure 2)

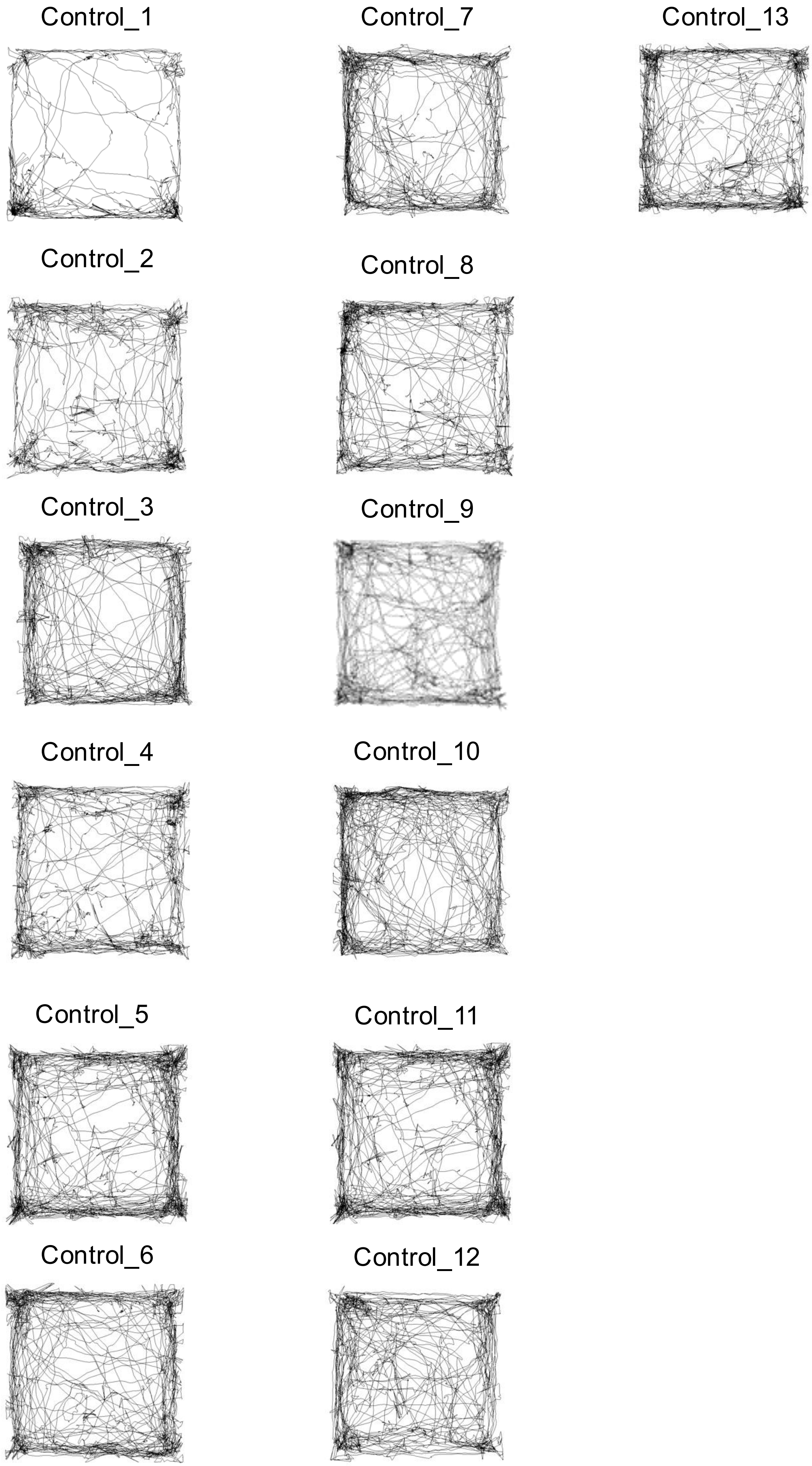

WT-CSDS KNT-127 for 14 days (related to Figure 4)

WT-CSDS Saline for 14 days (related to Figure 4)

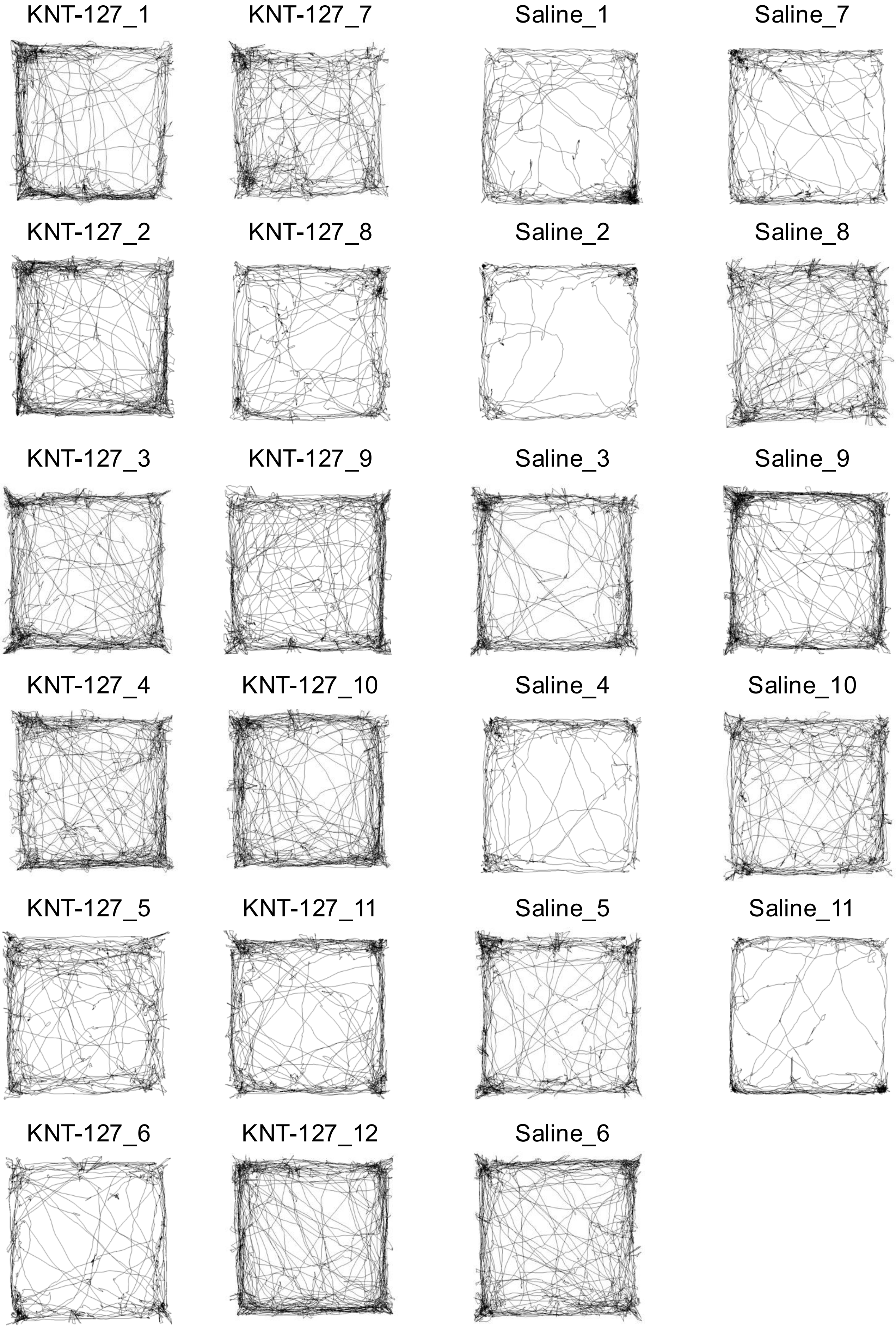

WT-CSDS After CSDS
(related to Figure 2, S4)

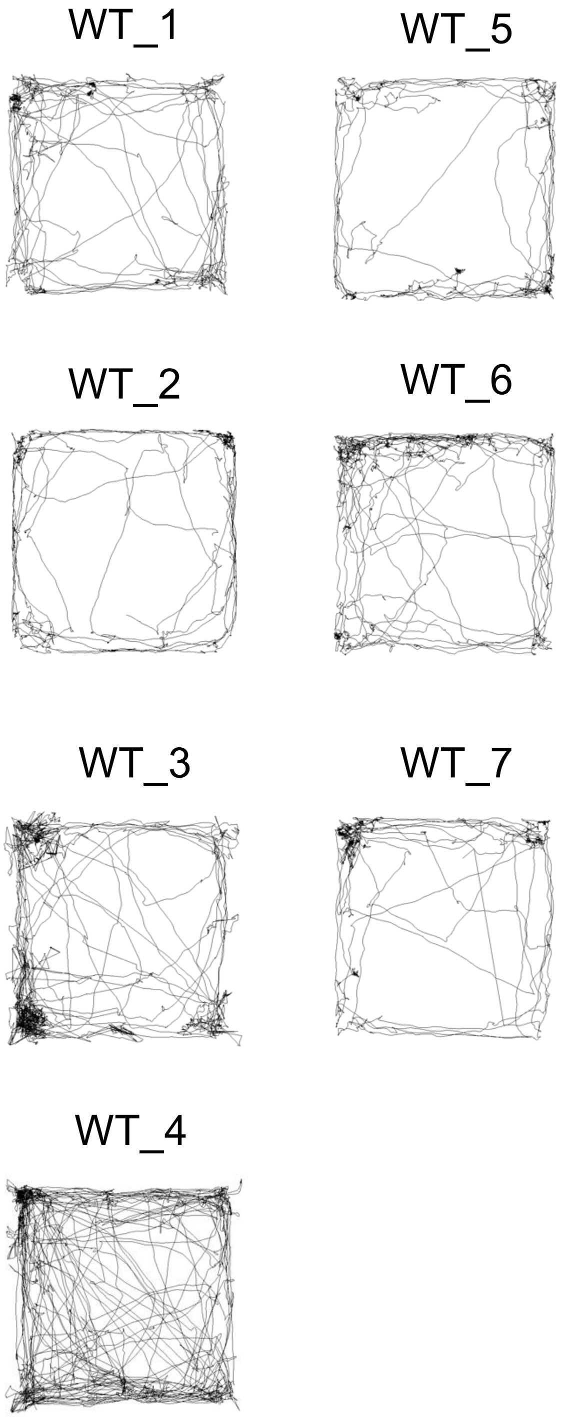

KO-CSDS After CSDS
(related to Figure 2, S4)

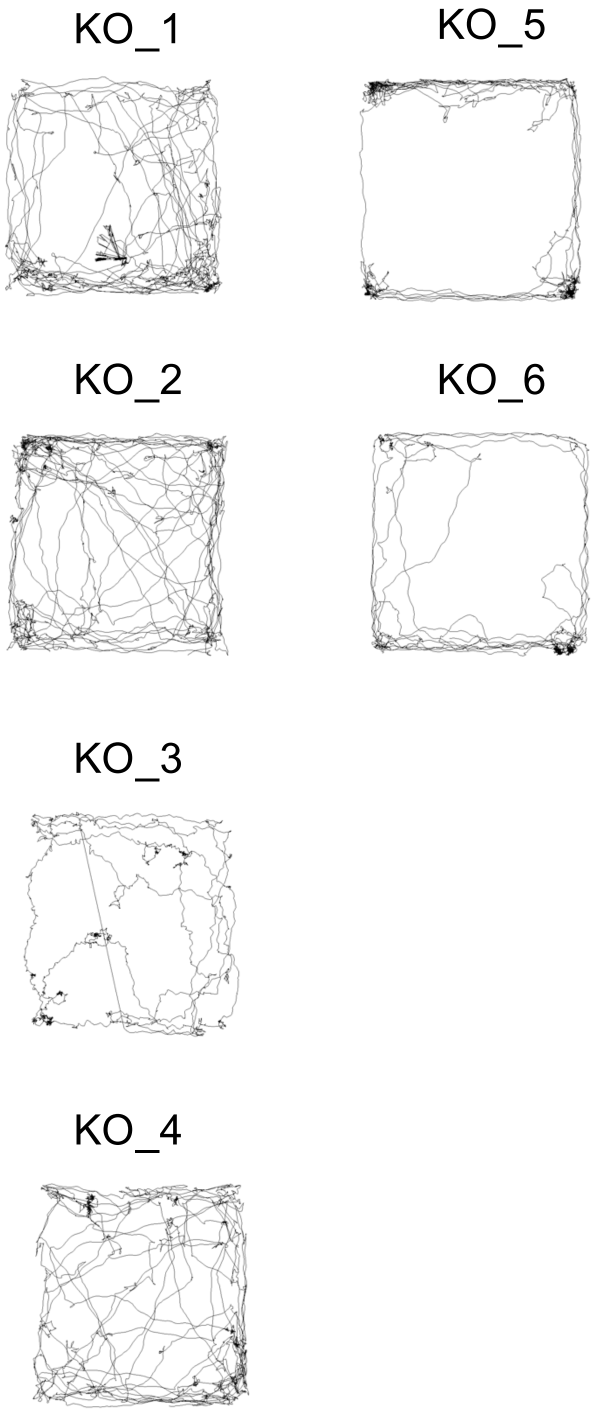

KO-Control After CSDS
(related to Figure 2, S4)

KO_1

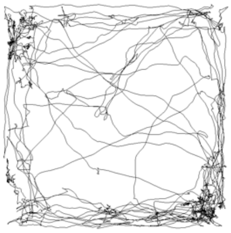

KO_2

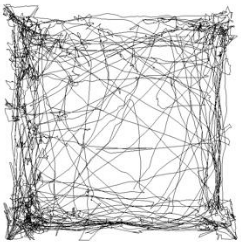

KO_3

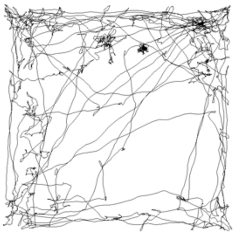

KO_4

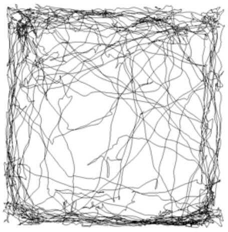

KO_5

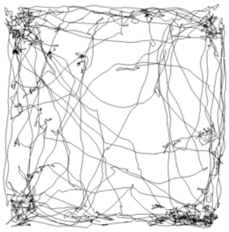

WT-Control After CSDS
(related to Figure 2, S4)

WT_1

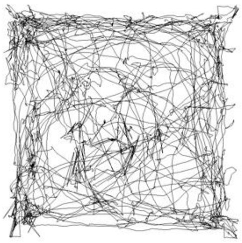

WT_2

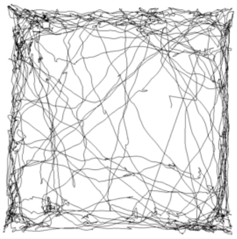

WT_3

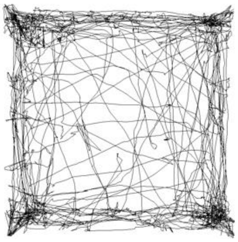

WT_4

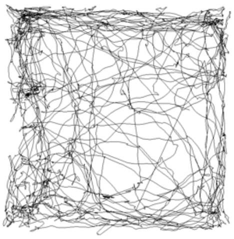

WT-CSDS
KNT-127 for 14days
(related to Figure 2, S4)

KO-CSDS
KNT-127 for 14days
(related to Figure 2, S4)

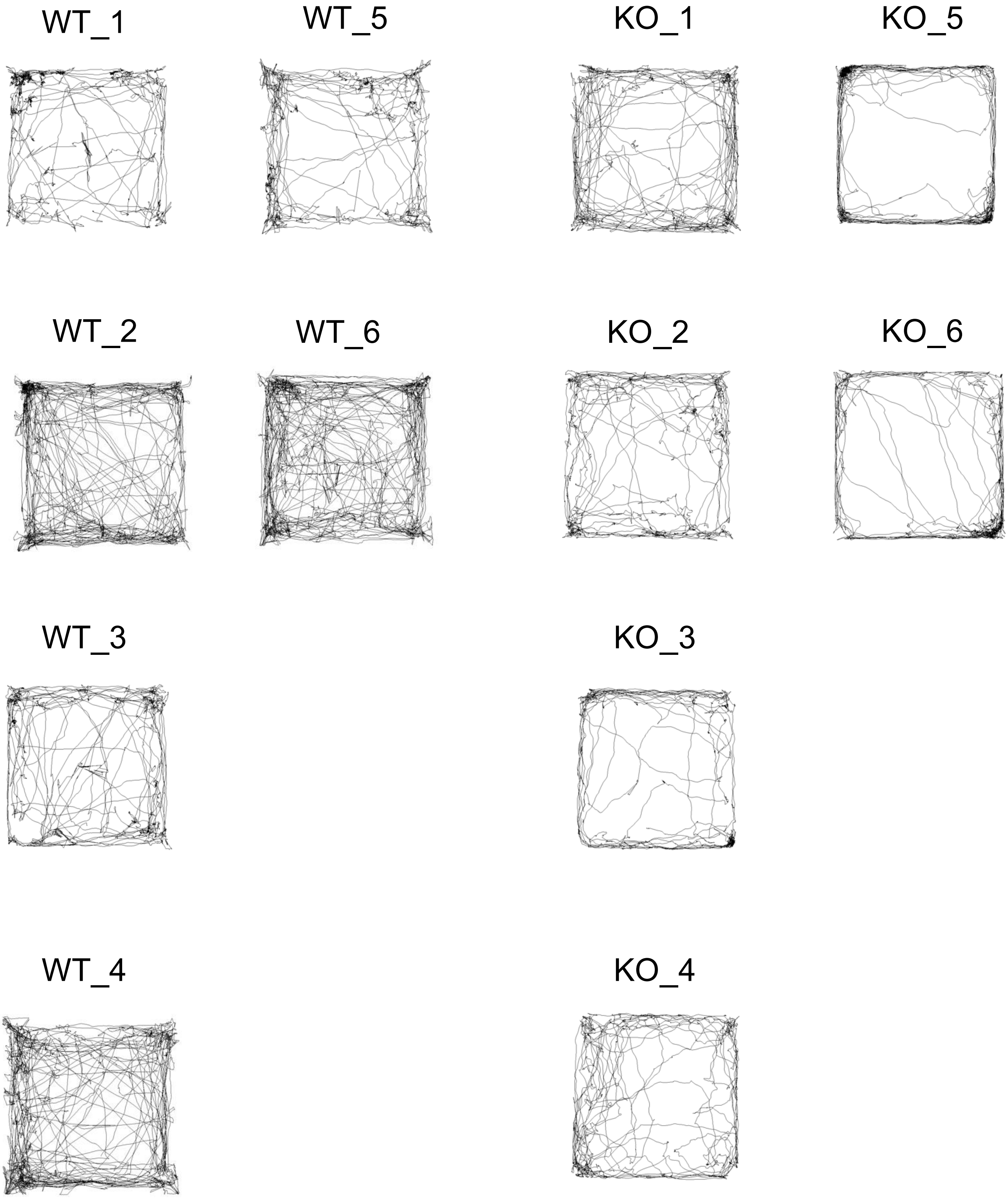

Supplement: Supplementary file 1 — Figure S1: Generation of Oprd1‐Cre knock‐in mice by CRISPR Cas9 system. Figure S2: Two clusters of CSDS mice can be identified on the basis of the time spent in the avoidance/interaction zone in the SIT, both before and after CSDS (related to Figure 1). Figure S3: Histogram showing the distribution of SI scores in CSDS mice and control mice (related to Figures 1 and 5). Figure S4: Social avoidance, anxiety, or depression‐like behaviors induced by CSDS in WT or DOP KO mice (related to Figure 4). [file NPR2-45-e70059-s001.zip › npr270059-sup-0001-DataS1.pdf]
