# Supplementary material for: Rapid‐Onset Therapeutic Effects of Delta Opioid Receptor Agonists on Depression‐Like Behaviors Induced by Chronic Social Defeat Stress
Source: Neuropsychopharmacol Rep. 2025 Sep 29;45(4):e70059. doi: 10.1002/npr2.70059 (PMC12479375; doi:10.1002/npr2.70059)
Supplement: Supplementary file 1 — Figure S1: Generation of Oprd1‐Cre knock‐in mice by CRISPR Cas9 system. Figure S2: Two clusters of CSDS mice can be identified on the basis of the time spent in the avoidance/interaction zone in the SIT, both before and after CSDS (related to Figure 1). Figure S3: Histogram showing the distribution of SI scores in CSDS mice and control mice (related to Figures 1 and 5). Figure S4: Social avoidance, anxiety, or depression‐like behaviors induced by CSDS in WT or DOP KO mice (related to Figure 4). [file NPR2-45-e70059-s001.zip › npr270059-sup-0003-FiguresS1-S4.docx]

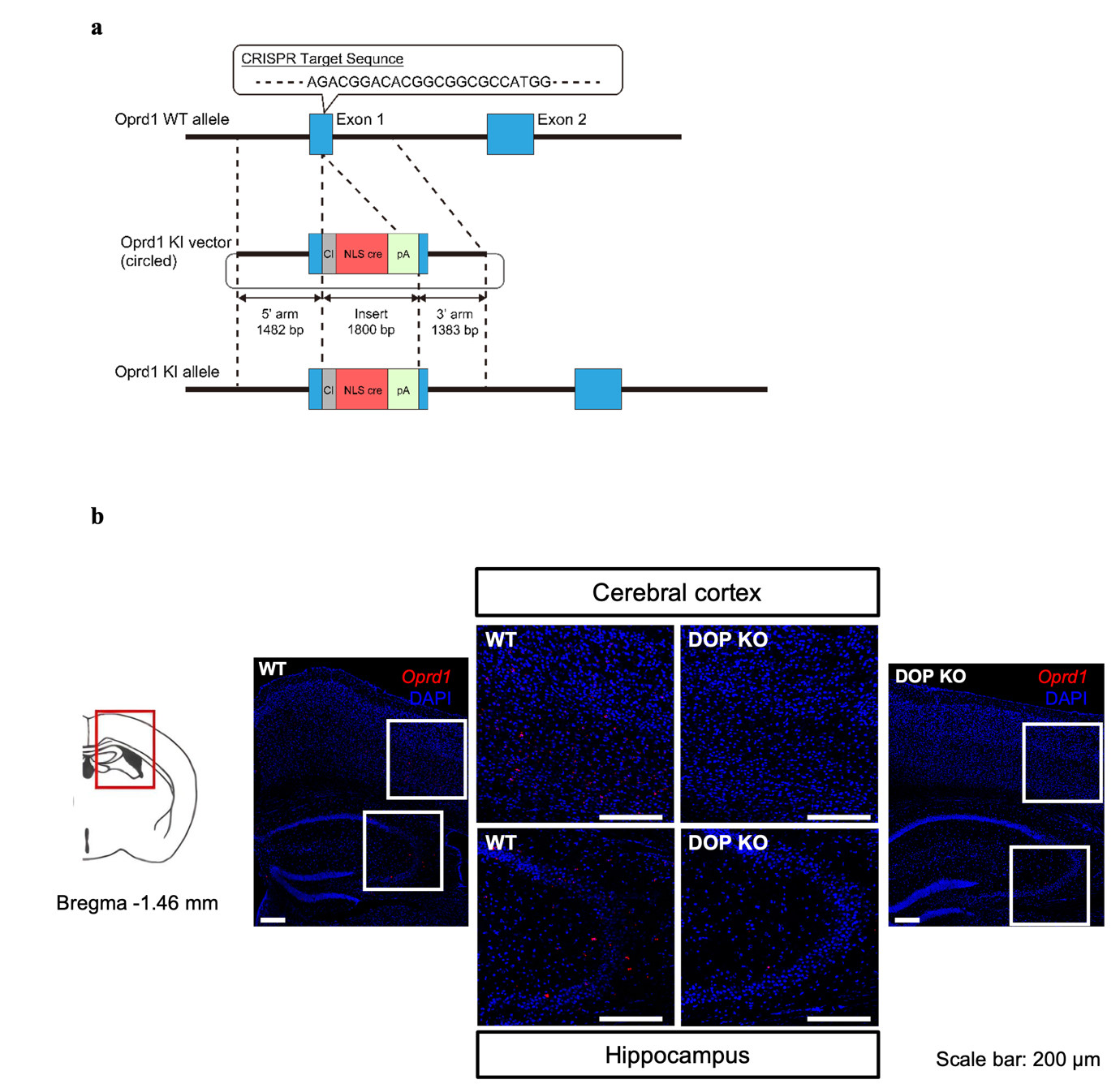


**Figure S1. Generation of Oprd1-*Cre* knock-in mice by CRISPR Cas9 system**

(a) Knock-in construct and targeting strategy for the *Oprd1* allele. CI, chimeric intron. NLS, nuclear translocation signal. pA, rabbit globin polyadenylation sequences. (b) Representative images demonstrating *Oprd1* mRNA expression in the hippocampus and cerebral cortex of wild-type and homozygous *Oprd1*-cre mice (also referred to as DOP KO mice).

**Figure S2. Two clusters of CSDS mice can be identified on the basis of the time spent in the avoidance/interaction zone in the SIT, both before and after CSDS (related to Figure 1)**

Two clusters of CSDS mice on the basis of the time spent in the avoidance or interaction zone in the SIT, both before and after CSDS (related to Figure 1c). Cluster A (blue) includes 47 mice from the mice after CSDS and 5 mice from the mice before CSDS. The cluster B (orange) includes 51 mice from the mice before CSDS and 1 mouse from the mice after CSDS.

**Figure S3. Histogram showing the distribution of SI scores in CSDS mice and control mice (related to Figure 1 and 5)**

A total of 71 CSDS-exposed mice and 24 control mice were classified on the basis of SI scores. The SI score of control mice averaged 88.50 ± 14.74 (mean ± SD) and ranged from 72.9 to 138.2. The cutoff criterion was set at an SI score < 70, a value that falls well below the minimum observed in control mice.

**Figure S4. Social avoidance, anxiety or depression-like behaviours induced by CSDS in WT or DOP KO mice (related to Figure 4)**

(a) Violin plots showing the mean time in the center zone (left) and total distance traveled (right) by WT (control, n = 4 and CSDS, n = 7) and DOP KO (control, n = 5 and CSDS, n = 6) mice in the OFT. *p < 0.05, **p < 0.01, ***p < 0.001 compared to control WT mice, ##p < 0.01 compared to control DOP KO mice, †p < 0.05 compared to CSDS DOP KO mice. one-way ANOVA followed by Tukey’s test. (b) Violin plot showing the mean immobility time of WT (control, n = 4 and CSDS, n = 7) and DOP KO (control, n = 5 and CSDS, n = 6) mice in the TST. Data represent mean ± SEM. *p < 0.05 compared to control WT mice, #p < 0.05 compared to control DOP KO mice, †p < 0.05 compared to CSDS DOP KO mice. one-way ANOVA followed by Tukey’s test. (c) Changes in social interaction (SI) score before and after CSDS in WT (left, n = 7) and DOP KO (right, n = 6) mice *p < 0.05, **p < 0.01, paired Student’s *t*-test.
